# Bridging molecular and heterogeneous catalysis: metallolinker-assembled and post-synthetically metalated covalent organic frameworks for photocatalytic CO_2_ reduction

**DOI:** 10.3389/fchem.2026.1976872

**Published:** 2026-10-12

**Authors:** Yuan-Hui Zhong, Lai-Hon Chung

**Affiliations:** 1 School of Chemistry and Environment, Jiaying University, Meizhou, Guangdong, China; 2 School of Chemical Engineering and Light Industry, Guangdong University of Technology, Guangzhou, Guangdong, China

**Keywords:** CO_2_ reduction, covalent organic framework, heterogeneous catalysis, metallolinker, photocatalysis

## Abstract

Photocatalytic carbon dioxide reduction (PCO_2_RR) represents a promising approach for solar-to-chemical energy conversion and carbon utilization, yet catalyst development remains limited by the trade-off between the molecular precision of homogeneous complexes and the robustness of heterogeneous materials. Metallolinker-assembled covalent organic frameworks (COFs) address this challenge by integrating well-defined metal coordination sites into crystalline porous architectures, combining tunable catalytic environments with framework stability and recyclability. Beyond acting as catalytic centers, metallolinkers regulate light harvesting, charge separation, CO_2_ adsorption, and local reaction environments through synergistic interactions with the surrounding framework. This review highlights recent advances in metallolinker-assembled COFs for PCO_2_RR and, for direct comparison, in the related family of post-synthetically metalated COFs, emphasizing how coordination chemistry, electronic structure, topology, and pore microenvironments govern catalytic activity and product selectivity. The review discusses key strategies, including metal-site modulation, linker engineering, donor–acceptor coupling, dual-site construction, and microenvironmental regulation, to elucidate structure–function relationships in CO_2_ activation and conversion. Remaining challenges, including operational stability, sacrificial-agent-free systems, C_2+_ product formation, mechanistic understanding, and scalable fabrication, are critically examined. Emerging opportunities in computational design, operando characterization, multifunctional frameworks, and device integration are further discussed to advance metallolinker-COFs toward practical solar-driven carbon conversion technologies.

## 1 Introduction

Photocatalytic CO_2_ reduction (PCO_2_RR) has emerged as a leading strategy for sustainable carbon valorization, reconciling rising atmospheric CO_2_ levels with the global transition to carbon-neutral energy systems (Hepburn et al., 2019). The advantages of PCO_2_RR over electrochemical CO_2_ reduction lie not in absolute reaction rate but in system simplicity and energy autonomy: (i) solar photons are used directly, without an intermediate electricity-generation and transmission step; (ii) no external power supply, ion-conducting electrolyte, supporting salt, membrane, or gas-diffusion electrode is required, which removes a substantial part of the balance-of-plant cost and enables compact, low-maintenance reactor configurations; (iii) the reaction proceeds at ambient temperature and pressure; and (iv) the technology is intrinsically modular and therefore well suited to decentralized deployment and to the direct utilization of dilute or impure CO_2_ streams (Wu et al., 2022; Singh et al., 2024). These features make PCO_2_RR a complementary rather than a competing route to electrolysis: electrolysis currently delivers far higher areal rates (partial current densities of hundreds of mA·cm^−2^), whereas photocatalysis offers the prospect of a self-contained, infrastructure-lean solar-to-fuel platform. Most critically, when the oxidative half-reaction is driven by H_2_O oxidation rather than by a sacrificial donor, photocatalytic systems can in principle close the carbon and electron (e^−^) cycles by converting only earth-abundant CO_2_ and water into value-added carbon-based fuels including CO, HCOOH, CH_4_, C_2+_ oxygenates (Fang et al., 2023; Gao et al., 2024).

The key to achieving PCO_2_RR is rational design of high-performance photocatalysts, which demands a synergistic integration of three key attributes: a high density of accessible catalytic sites to drive surface reactions, exceptional structural robustness to ensure longevity across multiple catalytic cycles, and crystallographic precision to elucidate unambiguous structure–activity relationships (Mohata et al., 2025; Zhou et al., 2022; Yang et al., 2024). However, the design of efficient photocatalysts remains fundamentally constrained by a persistent trade-off: homogeneous molecular complexes offer atomically defined, tunable coordination environments that enable high product selectivity, yet suffer from poor operational stability, challenging recovery, and aggregation-induced deactivation; heterogeneous semiconductors provide superior structural durability and recyclability, but their ill-defined active sites lead to ambiguous reaction mechanisms and limited selectivity control (Zhang et al., 2020).

Covalent organic frameworks (COFs), crystalline porous polymers linked by strong covalent bonds, provide a platform to integrate these complementary attributes (Basak et al., 2023; Rodríguez-Camargo et al., 2024; Liu G-F. et al., 2025). Throughout this review, the term “metallolinker” denotes a molecular building block in which a catalytically relevant metal center is held by a well-defined chelating site (Diercks et al., 2018; Kim M. et al., 2023), so that the metal is installed at the monomer stage and retained within the framework after polymerization (Chen et al., 2023; Keller and Bein, 2021; Bag et al., 2023). COFs assembled from such pre-metalated linkers—metallolinker COFs—are the primary subject of Section 3.1. COFs in which the chelating site is built into the framework, and the metal is introduced afterward, are termed post-synthetically metalated COFs; because they do not satisfy the definition above but generate the same type of atomically dispersed metal site, they are discussed in Section 3.2 as a family for direct comparison.

## 2 Fundamental concepts and principles of PCO_2_RR

### 2.1 Thermodynamics and reaction pathways of PCO_2_RR

Photocatalytic CO_2_ reduction is a proton-coupled, multielectron-transfer (PCET) process that converts thermodynamically stable CO_2_ into C_1_ products, including CO, HCOOH, HCHO, CH_3_OH and CH_4_, as well as C_2+_ products such as C_2_H_4_ and C_2_H_5_OH (Dey et al., 2026). The difficulty of this transformation stems from the exceptional kinetic stability of CO_2_, arising from its linear $D_{\infty h}$ geometry, short C=O bonds (∼1.16 Å) and high bond strength (∼750 kJ·mol^−1^) (Zhong et al., 2024). Direct one-electron reduction to CO_2_^•−^ is particularly demanding, with a commonly cited potential of approximately −1.90 V *vs*. SHE at pH = 7, because adding an e^−^ requires substantial molecular reorganization from linear CO_2_ toward a bent radical-anion geometry. Photocatalytic systems therefore generally proceed through proton-assisted multielectron pathways, in which PCET avoids accumulation of the high-energy CO_2_^•−^ intermediate and reduces the energetic penalty associated with individual reaction steps. The standard reduction potentials of the principal CO_2_ reduction pathways at pH 7 are summarized in Table 1. The overall concept of metallolinker-based COFs for PCO2RR is illustrated in Scheme 1.

**TABLE 1:** The standard reduction potentials for the major CO_2_RR pathways (Dey et al., 2026).

Reaction	*E* ^0^ (V *vs*. SHE, pH = 7)
2H^+^ + 2e^−^ → H_2_ (HER)	−0.41
CO_2_ + e^−^ → CO_2_^•−^	−1.90
CO_2_ + 2H^+^ + 2e^−^ → HCOOH	−0.61
CO_2_ + 2H^+^ + 2e^−^ → CO + H_2_O	−0.53
CO_2_ + 4H^+^ + 4e^−^ → HCHO + H_2_O	−0.48
CO_2_ + 6H^+^ + 6e^−^ → CH_3_OH + H_2_O	−0.38
CO_2_ + 8H^+^ + 8e^−^ → CH_4_ + 2H_2_O	−0.24

**SCHEME 1:**
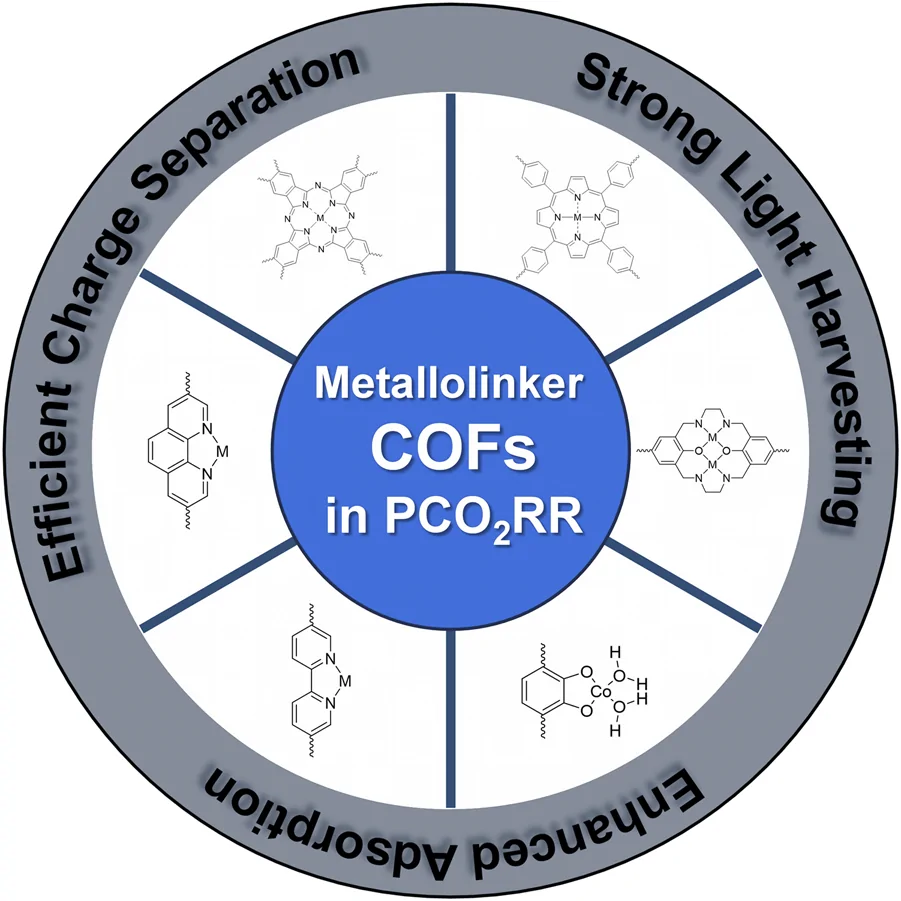
Illustration of metallolinker-based COFs for PCO_2_RR.

Two features of these potentials are particularly relevant to catalyst design. First, the thermodynamic potentials of several CO_2_ reduction products lie within a relatively narrow range and overlap with that of the competing hydrogen evolution reaction (HER; *E* ^0^ ≈ −0.41 V *vs*. SHE), making product selectivity strongly dependent on reaction kinetics and intermediate binding rather than on thermodynamic driving force alone (Rath et al., 2024). Second, products requiring more e^−^ are not intrinsically less accessible thermodynamically; for example, the formal reduction potential for CH_4_ formation is more positive than that for CO formation. Their lower formation rates instead reflect the greater kinetic complexity of successive PCET steps, the need to stabilize multiple surface-bound intermediates, and competition with HER (Chakraborty et al., 2026). Product distribution is therefore governed primarily by the adsorption geometry and relative stabilization of key intermediates at the catalytic center (Cao et al., 2025). Two major mechanistic branches are commonly distinguished. In the carboxyl pathway, adsorbed CO_2_ undergoes proton-coupled reduction to *COOH (alternatively denoted as *OCOH), which can yield CO after further reduction and desorption or proceed through *CO (Zhong et al., 2019), *CHO and related intermediates toward deeper reduction products such as CH_4_ (Biswas et al., 2024); sufficiently stabilized *CO intermediates may also undergo C–C coupling to generate C_2+_ products (Xu et al., 2026). Formation of *COOH is frequently a major kinetic barrier for CO-producing pathways, although the rate-determining step (RDS) depends on the catalyst and reaction environment. In the HCOOH pathway, CO_2_ is converted to an O-bound *OCHO intermediate (Wang J.M. et al., 2023), either through interaction with a metal hydride or through PCET, favoring HCOOH formation and often becoming competitive on relatively oxophilic metal centers.

A viable photocatalyst must consequently provide both appropriate redox energetics and favorable reaction kinetics: its e^−^-accepting states, often described in COFs in terms of the conduction-band (CB) edge or lowest-unoccupied molecular orbital (LUMO)-derived states, must be sufficiently reducing to drive the desired CO_2_ conversion, whereas its hole (*h* ^+^)-accepting states must be sufficiently oxidizing to regenerate the photocatalyst through oxidation of H_2_O or a sacrificial donor such as triethanolamine (TEOA) (Lan et al., 2024; Sun et al., 2026). Band alignment alone, however, does not determine activity. The markedly more negative potential associated with isolated CO_2_^•−^ formation relative to proton-assisted CO_2_-to-CO conversion highlights the importance of stabilizing bent, activated CO_2_ and protonated intermediates at the catalytic site (Wang S. et al., 2023; Yin et al., 2024).

### 2.2 Critical steps in the photocatalytic process

Whereas thermodynamics defines the accessible pathways of PCO_2_RR, the overall photocatalytic performance is governed by a sequence of kinetically coupled processes—light absorption and exciton generation, charge separation and migration, CO_2_ adsorption and activation, surface catalytic conversion, and consumption of photogenerated holes as schematically illustrated in Figure 1 (Yin et al., 2026). Losses at any stage can limit the overall quantum efficiency or alter product selectivity. (i) *Light absorption and exciton generation*: Photocatalysis commences with photon absorption energetic enough to access excited states of the framework. Efficient catalysis demands not only broad light absorption but also excited states with appropriate energies and lifetimes to enable subsequent charge separation and interfacial electron transfer (Kuramochi et al., 2018). (ii) *Charge separation and migration*: Productive charge separation must outcompete radiative and non-radiative relaxation. In molecular photocatalysts, short-lived excited states limit intermolecular e^−^ transfer; in heterogeneous materials, recombination may occur in the bulk, at interfaces, or via defect states. e^−^ and h^+^ must reach reduction and oxidation sites before recombining (Chang et al., 2016). (iii) *Substrate adsorption and activation*: CO_2_ reduction requires adequate substrate availability at the catalytic site and stabilization of an activated adsorption geometry (Rong et al., 2025). (iv) *Surface catalytic conversion*: At the catalytic center, CO_2_ reduction proceeds through PCET whose energetics depend on the metal identity, coordination geometry and surrounding chemical environment. Product selectivity reflects the relative binding energies and kinetic accessibility of intermediates. Rational control of intermediate binding is therefore central to directing the reaction toward two-electron products or more deeply reduced C_1_ and C_2+_ products (Han et al., 2023). (v) *Hole scavenging*: Sustained photocatalysis requires rapid consumption of photogenerated h^+^. Most reported systems employ sacrificial electron donors (*e.g.*, TEOA or ascorbic acid) that undergo irreversible oxidation, suppressing charge recombination while keeping the catalyst in a reduced, active state. Although convenient for mechanistic studies, this approach precludes a complete solar-fuel cycle (Gong et al., 2023).

**FIGURE 1:**
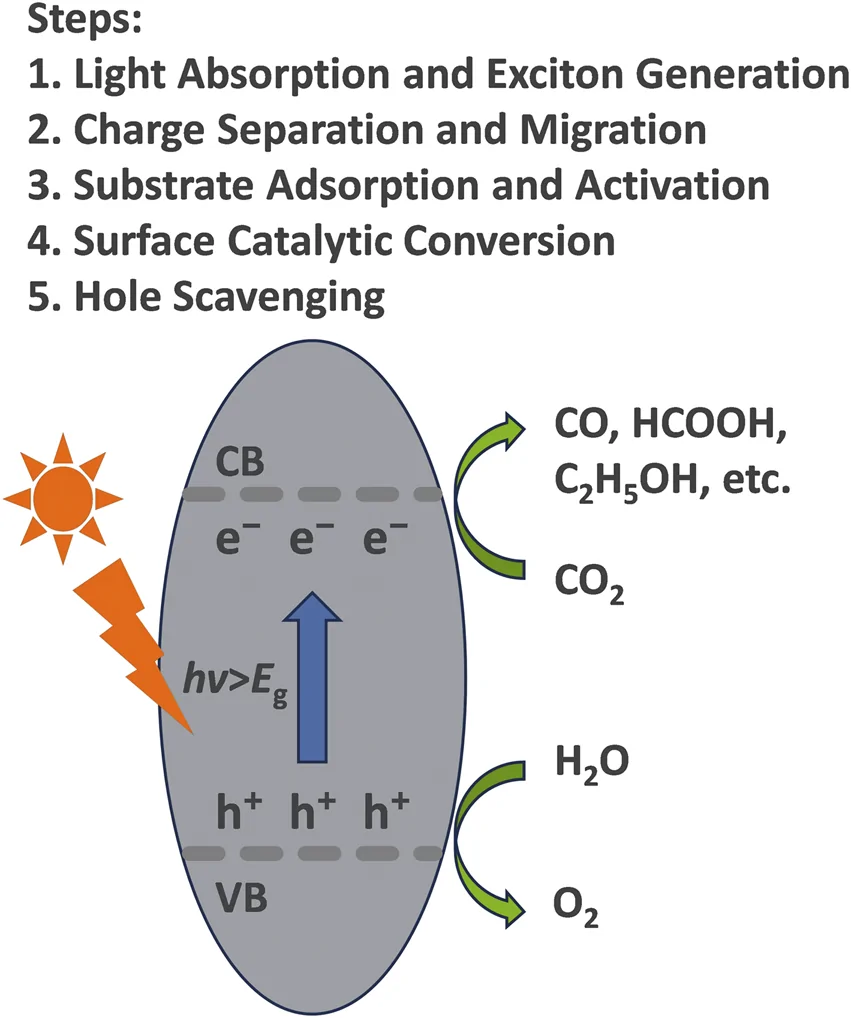
Illustration of mechanism for PCO_2_RR.

### 2.3 Challenges of photocatalytic CO_2_ reduction

While the sequential kinetic steps outlined above define the idealized mechanistic blueprint for efficient PCO_2_RR, translating this framework into practical, scalable catalytic performance remains hindered by persistent, interrelated bottlenecks across thermodynamic, kinetic, materials, and operational dimensions.

#### 2.3.1 Product selectivity

CO_2_ is chemically inert with a high C=O bond energy of ∼750 kJ·mol^−1^, and its one-electron reduction to CO_2_^•−^ requires a highly negative potential due to the large reorganization energy associated with bending the linear CO_2_ molecule (Li et al., 2019). While PCET lowers the effective reduction potential to a range accessible to many photocatalysts, the thermodynamic potentials for reducing CO_2_ to value-added products (CO, HCOOH, HCHO, CH_3_OH, CH_4_) cluster within a narrow window of −0.7 to −0.2 V *vs*. SHE at pH = 7 (Albero et al., 2020; Tomboc et al., 2020). This minimal thermodynamic discrimination between products, coupled with ambiguous multi-step PCET mechanisms, often leads to product mixtures that are difficult to separate based on physicochemical properties (Kovačič et al., 2020). Five elements are decisive. (i) *The first protonation step sets the branch*: once CO_2_^•−^ coordinates bidentate to the metal, protonation at carbon gives O-bound *OCHO and thus HCOOH (Yadav et al., 2016), whereas protonation at oxygen gives *COOH and terminates in CO (Biswas et al., 2023); the outcome tracks the oxophilicity of the metal center. (ii) *Proton activity must be matched to electron delivery rather than maximized*: in a bipyridine-based Cu COF, CH_4_ is obtained in water whereas >90% CO is obtained in DMF (Zhang Y. et al., 2023), indicating that media rich in e^−^ and H* favor full deoxygenation, because protonation of *CO to *CHO is the RDS toward CH_4_. (iii) Retention versus full deoxygenation of *CO: *CO must bind strongly enough to be hydrogenated yet weakly enough for the pathway to stop at six e^−^; whether *CH_3_ meets *OH or *H decides CH_3_OH versus CH_4_. (iv) *Liquid products must be protected once formed*: since photogenerated h^+^, O_2_ and H_2_O_2_ re-oxidize them more readily than gaseous CO or CH_4_ (Biswas et al., 2024; Fu et al., 2018); high pH, hydrophobic microenvironments and weakly hydrogen-bonding solvents therefore improve apparent CH_3_OH selectivity. (v) *For C*_*2+*_ *liquid fuels, an additional and considerably stricter structural requirement appears*: C_2+_ liquids additionally require two adjacent intermediates able to dimerize, which single sites stabilize poorly (Huang et al., 2025; Yang et al., 2023). Finally, because HCOOH, CH_3_OH and higher oxygenates can also originate from decomposition of the framework, solvent or sacrificial donor, products should be verified by ^13^CO_2_ labeling, carbon balance, and dark and Ar controls, and selectivity reported on an electron basis.

A separate and equally decisive variable, distinct from the reaction-level factors (i)–(v) above, is the electronic structure of the metal center itself, which governs how strongly the key intermediates are bound and therefore how far the reduction proceeds (Zhang et al., 2024). Three coupled descriptors can be distinguished. (a) *Metal identity sets the intrinsic binding preference, largely through the position of the metal d-band relative to the adsorbate Frontier orbitals*: relatively oxophilic or e^−^-poor centers (*e.g.*, Co^III^, Ni, Fe) favor O-bound *OCHO and direct the reaction towards HCOOH or CO (Yadav et al., 2016), whereas e^−^-rich centers able to bind C-bound intermediates more strongly promote deeper reduction to CH_4_ (Zhang Y. et al., 2023). A change of oxidation state at the same metal in the same lattice can therefore invert product selectivity. (b) *Oxidation state modulates both the adsorption strength and the elementary-step barrier*: for the same porphyrinic framework, Co^III^–N_4_ increases the calculated Co–O adsorption energy from 0.05 to 0.11 eV and shortens the Co–O distance from 2.82 to 2.31 Å relative to Co^II^–N_4_, lowering the barrier for HCOOH formation from 1.32 to 0.68 eV while raising that for the subsequent conversion of HCOOH to CO or CH_4_ from 0.97 to 1.20 eV. A change of oxidation state at the same metal in the same lattice can therefore invert product selectivity (Gong et al., 2020). (c) *Spin state is a further descriptor whose effect is difficult to separate from that of oxidation state*: the reported Co^II^ (*S* = 1/2) and Co^III^ (*S* = 0) frameworks also differ in which Co 3*d* orbital couples most strongly to adsorbed CO_2_, so the two contributions cannot be disentangled from the available data alone (Gong et al., 2020). Because these variables are usually varied together, unambiguous structure–selectivity correlations require isostructural series in which only one parameter is changed, combined with operando X-ray absorption spectroscopy and computational analysis of the full free-energy landscape.

#### 2.3.2 Parasitic hydrogen evolution reaction (HER)

Liquid-phase PCO_2_RR typically uses H_2_O as the solvent to avoid toxic organic alternatives and provide a sustainable proton source for PCET (Ghosh et al., 2024). However, the reduction potential for H_2_ evolution (E(H_2_O/H_2_) = −0.41 V *vs*. SHE at pH = 7) falls squarely within the narrow potential window for CO_2_ reduction products, and the HER is kinetically favored as a two-electron process compared to the multi-electron transfer required for most CO_2_ reduction products (Kuriki et al., 2015). This thermodynamic and kinetic favorability is further exacerbated by the low solubility of CO_2_ in H_2_O, with only 1 CO_2_ molecule per ∼1300 H_2_O molecules at 1 atm pressure (Li et al., 2018), meaning H_2_O is far more accessible to catalytic sites than CO_2_ under typical reaction conditions (Das et al., 2021; Tomita et al., 2000). The HER thus acts as a parasitic competing reaction that severely compromises the selectivity and solar-to-fuel efficiency of PCO_2_RR systems.

#### 2.3.3 Reliance on precious metals

Despite intensive efforts to develop high-performance PCO_2_RR photocatalysts, most state-of-the-art systems rely on transition metals with accessible multi-electron redox states as active sites. Many also require cocatalysts or external photosensitizers based on scarce, expensive noble metals (*e.g.*, Ru, Ir) to improve light harvesting or charge-transfer efficiency (Tamaki and Ishitani, 2017). This reliance on costly, low-abundance metals poses a major barrier to scalability and undermines the sustainability of the overall process. Transition metal-based systems also face additional limitations: they are prone to self-oxidation, photocorrosion, and poor hydrolytic stability, and often exhibit unintended selectivity toward the HER (Shen et al., 2020; Xie et al., 2023). Metal-free alternatives have been explored to address these issues, but they typically suffer from lower activity and selectivity than their metal-containing counterparts.

#### 2.3.4 Oxidation half-reaction

The holes generated in the photocatalyst valence band during illumination must be rapidly consumed to prevent charge recombination and maintain catalytic turnover (Guo et al., 2019). While the valence band edges of most semiconductor photocatalysts are sufficiently positive (E(O_2_/H_2_O) = 0.81 V *vs*. SHE at pH = 7) to thermodynamically drive H_2_O oxidation to O_2_, this four-electron process is kinetically sluggish and often leads to the accumulation of highly oxidizing intermediates that can degrade the photocatalyst itself (Verma et al., 2022). To bypass this kinetic bottleneck, most reported systems rely on sacrificial electron donors (SEDs) such as TEOA or ascorbic acid, which irreversibly consume holes but add cost, introduce secondary waste streams, and mask the true efficiency of the oxidation half-reaction, preventing a closed-loop, net-energy-gain solar fuel cycle (Qu et al., 2024; Zhou et al., 2020).

#### 2.3.5 Catalyst decomposition under operational conditions

Operational stability remains a major unresolved challenge for all classes of PCO_2_RR photocatalysts. For example, graphitic carbon nitride (*g*-C_3_N_4_), a widely studied organic photocatalyst, undergoes self-decomposition under illumination, generating CO, CO_2_, and nitrogenous byproducts by cleaving C–N=C bonds activated by adsorbed hydroxyl groups (Chen et al., 2022). While the photostability of conventional semiconductors is well characterized, systematic studies of photocatalyst degradation mechanisms remain scarce. This knowledge gap represents a critical barrier to the practical deployment of PCO_2_RR systems.

### 2.4 Key physicochemical attributes of high-performance catalysts for PCO_2_RR

PCO_2_RR catalysts require a delicate balance of molecular precision and bulk material properties to overcome the inherent kinetic and thermodynamic barriers of the reaction. Ideal photocatalysts combine the well-defined active sites of homogeneous systems with the structural robustness and recoverability of heterogeneous materials (Gao et al., 2017). By integrating pre-engineered catalytic motifs into tailored architectures, these systems can simultaneously regulate catalytic-site structure, light harvesting, charge separation and transport, substrate enrichment, and the local reaction environment, thereby addressing several limitations that govern PCO_2_RR efficiency.

#### 2.4.1 Atomically precise and tunable electronic structures

A defining feature of advanced PCO_2_RR catalysts is the ability to preserve molecularly defined coordination environments within a functional scaffold. Unlike the heterogeneous and often poorly resolved surface sites of conventional semiconductors, well-designed catalysts provide distributed active centers with specific coordination geometries in a periodic manner. This structural uniformity enables precise control over the electronic properties of the metal center (Luo et al., 2024). Modifying the ligand scaffold or its secondary coordination environment can adjust metal oxidation and spin states, ligand-field strength, orbital energies, and axial coordination. Such changes directly affect CO_2_ adsorption and the relative stabilization of key reaction intermediates (Zhang et al., 2024; Li et al., 2026). Consequently, high-performance photocatalysts provide a platform to systematically link catalytic selectivity to coordination structure and electronic configuration, moving catalyst design beyond empirical optimization toward mechanistically informed structure–activity relationships.

#### 2.4.2 Enhanced light harvesting and excited-state energy transfer

Catalytic precision must be complemented by efficient utilization of incident light. Catalysts incorporating chromophores extend strongly into the visible region, enabling broader solar-spectrum absorption than many wide-band-gap inorganic photocatalysts (Yang et al., 2025). Within an ordered porous framework, these chromophores interact electronically with surrounding π-conjugated linkers rather than behaving as isolated molecular absorbers (Cappelletti et al., 2024; Chen et al., 2024). When spectral overlap, intermolecular distance, and dipole orientation are optimized, excitation energy generated on peripheral organic units can transfer to catalytic sites via Förster resonance energy transfer (FRET) (Han et al., 2025). This antenna-like pathway concentrates excitation near the active center, increasing the probability that absorbed photons generate productive charge-separated states.

#### 2.4.3 Efficient charge separation and extended carrier lifetimes

Photogenerated charge separation represents a critical advantage when the electronic structure of the catalytic site and the supporting framework are appropriately matched. In many donor–acceptor (D–A) type photocatalysts, photoexcitation drives e^−^ transfer from the conjugated organic framework toward metal-centered or metal–ligand acceptor states, while h^+^ remain localized on the organic scaffold (Liu et al., 2024). This spatial separation suppresses radiative and non-radiative recombination, extending the lifetime of catalytically relevant charge-separated states. In optimized systems, charge-carrier lifetimes increase markedly compared to metal-free analogs, though observed timescales range from picoseconds to nanoseconds (or longer) depending on composition, orbital alignment, stacking, and defect density; a universal picosecond-to-microsecond transition should not be assumed. The crystalline and π-conjugated framework further supports directional charge migration via through-bond and through-space electronic coupling (Fu et al., 2026). In principle, arranging redox-active units with progressively offset potentials establishes an internal energetic gradient that promotes spatially directed transport of e^−^ and h^+^. Immobilization of catalytic sites within the framework provides an additional kinetic advantage: because active complexes are covalently fixed rather than freely diffusing, bimolecular back-electron-transfer reactions between oxidized photosensitizers and reduced intermediates are reduced relative to homogeneous multicomponent systems (Fukuzumi et al., 2018).

#### 2.4.4 Localized CO_2_ preconcentration and tailored microenvironments

Beyond electronic control, advanced photocatalysts provide a confined reaction environment that locally regulates CO_2_ adsorption, activation, and mass transport. Nanometer-scale pores enrich CO_2_ relative to the surrounding medium, increasing substrate availability near catalytic centers, a feature particularly valuable for dilute gas streams (Wang et al., 2024a). Catalysts with coordinatively unsaturated or Lewis-acidic metal centers further promote CO_2_ adsorption, polarization, and bending, stabilizing the activated geometries required for the initial e^−^-transfer or PCET step (Han et al., 2018). The secondary coordination sphere offers additional control: hydrophobic groups positioned near the active center reduce local H_2_O activity, limiting competitive HER and improving selectivity toward CO_2_-reduction products (Liu J. et al., 2025). However, such microenvironmental effects must be balanced with proton availability, as excessive H_2_O exclusion may impede PCET steps. Hierarchical porosity further optimizes mass transport: mesopores (2–50 nm) combined with intrinsic micropores facilitate diffusion of SEDs, reactants, and gaseous products while reducing pore blockage (Lv et al., 2023). This integration of molecularly defined active sites and spatially engineered transport pathways provides microenvironmental control unattainable with conventional homogeneous catalysts or porous solids alone.

#### 2.4.5 Comparative overview of representative photocatalysts for PCO_2_RR

Table 2 summarizes representative metallolinker-assembled COFs, post-synthetically metalated COFs, and other heterogeneous photocatalysts reported for PCO_2_RR, along with the nature of the metal site, irradiation conditions, photosensitizer, sacrificial donor, products formed, production rate, selectivity, and, where reported, apparent quantum yield. Because reported rates depend strongly on lamp power and spectral distribution, reactor geometry, catalyst loading and the presence of photosensitizers or cocatalysts, values obtained in different laboratories are not directly comparable and are listed for qualitative comparison only.

**TABLE 2:** Comparison of metallolinker-assembled COFs, post-synthetically metalated COFs, and representative inorganic and MOF-based photocatalysts for PCO_2_RR.

Photocatalysts	Metal/Coordination environment	Light source	Photosensitizer	Sacrificial agent	Product	Production rate	Selectivity	AQY	References
TTCOF-Zn	Zn–N_4_	420–800 nm	-	H_2_O	CO	12.33 μmol (cumulative)	100%	n.r.	Lu et al. (2019)
NiSN_3_Por-PDAN-COF	Ni–SN_3_	465 nm LED	Ru(bpy)_3_Cl_2_	BIH	CO	22.7 mmol·g^−1^·h^−1^	92.20%	n.r.	Wang et al. (2024c)
COF-367-Co^III^	Co–N_4_	300 W Xe lamp	-	TEA	HCOOH CO CH_4_	93 μmol·g^−1^·h^−1^ 5.5 μmol·g^−1^·h^−1^ 10.1 μmol·g^−1^·h^−1^	97.10%	n.r.	Gong et al. (2020)
Co-Por-BDT Co-Por-TT Co-Por-TA	Co–N_4_	150 W Xe lamp	Re(bpy)(CO)_3_Cl	TEOA	CO	1,424 μmol·g^−1^·h^−1^ 1,182 μmol·g^−1^·h^−1^ 642 μmol·g^−1^·h^−1^	∼94% ∼94% ∼94%	n.r.	Kim et al. (2023b)
EPCo-COF-AT EPCo-COF	Co-N_4_	300 W Xe lamp	Ru(bpy)_3_Cl_2_	TEOA	CO	17.7 mmol·g^−1^·h^−1^ 14.1 mmol·g^−1^·h^−1^	97.80% 85.1%	n.r.	Lin et al. (2024)
Co-DB-COF	Co–N_4_	Visible light (λ ≥ 420 nm)	Ru(bpy)_3_Cl_2_	TEOA	CO	25.7 mmol·g^−1^·h^−1^	92.30%	0.65% (450 nm)	Lin et al. (2025)
Co-TAPT-COF-1	Co–N_2_O_2_–Co	Visible light	Ru(bpy)_3_Cl_2_	TEOA	CO	8.39 mmol·g^−1^·h^−1^	42.59%	n.r.	Zhou et al. (2023)
ZnZn-Salen-COF	Zn–N_2_O_2_	Visible light (420 ≤ λ ≤ 800 nm)	-	H_2_O	CO	150.9 μmol·g^−1^·h^−1^	>99%	n.r.	Dong et al. (2025)
Cu-Bpy-COF	Cubpy(OAc)_2_	Visible light (λ > 420 nm)	-	TEA	CO CH_4_	40.8 μmol·g^−1^ (cumulative) 70.0 μmol·g^−1^ (cumulative)	100%	n.r.	Zhang et al. (2023a)
Co-Btt-Bpy COF	Co(bpy)	LED lamp (λ = 420 nm)	Ru(bpy)_3_Cl_2_	TIPA	CO	9,800 μmol·g^−1^·h^−1^	78.70%	1.10%	Xu et al. (2025)
K-Py-Bpy-COF-Co	Co(bpy)	LED lamp (400 ≤ λ ≤ 800 nm)	Ru(bpy)_3_Cl_2_	TEOA	CO	12,476.4 μmol·g^−1^·h^−1^	93.30%	2.8% (445 nm)	Song et al. (2025)
PY-CN-BIP-Ni	Ni–N_2_Cl_2_	Xe lamp (λ ≥ 420 nm)	-	BIH	CO	553.3 μmol·g^−1^·h^−1^	94%	0.82% (420 nm)	Qin et al. (2025)
Re-Bpy-sp^2^c-COF	Re(bpy)(CO)_3_Cl	300 W Xe lamp (> 420 nm)	-	TEOA	CO	1,040 μmol·g^−1^·h^−1^	81%	0.5% (420 nm)	Fu et al. (2020)
viCOF-bpy-Re	Re(bpy)(CO)_3_Cl	Xe lamp (> 420 nm)	-	H_2_O	CO	190.6 μmol·g^−1^·h^−1^	100%	1.08% (400 nm)	Cheng et al. (2023)
Co SAS/PAD-COF	Co–N_2_Cl_2_	Visible light	Ru(bpy)_3_Cl_2_	TEOA	CO	3,091 μmol·g^−1^·h^−1^	93%	n.r.	Ai et al. (2024)
Co-2,3-DHTA-COF	Co–O_4_	Visible light (λ ≥ 420 nm)	Ru(bpy)_3_Cl_2_	TEOA	CO	18,000 μmol·g^−1^·h^−1^	95.70%	0.47% (450 nm)	Zhang et al. (2023b)
Co-Oac	Co–N_3_OAc	300 W Xe lamp (> 380 nm)	-	BIH	CO	2,325.7 μmol·g^−1^·h^−1^	99.10%	n.r.	Sun et al. (2024)
NiTCPE-pstp	Ni_3_(OH)N_3_(COO)_6_	300 W Xe lamp	-	TIPA	CO	3,353.6 μmol·g^−1^·h^−1^	99.90%	n.r.	Zheng et al. (2023)
Au/ZTO	Zr/Ti bimetallic oxide	300 W Xe lamp	-	TEOA	C_2_H_4_/C_2_H_6_	6.80 μmol·g^−1^·h^−1^ 4.05 μmol·g^−1^·h^−1^	–	n.r.	Huang et al. (2024)

BIH: 1,3-dimethyl-2-phenyl-2,3-dihydro-1*H*-benzo[d] imidazole.

TIPA: triisopropanolamine.

Bpy/bpy: 2,2′-bipyridine.

TEOA, triethanolamine.

TEA: triethylamine.

“n.r.” denotes not reported.

## 3 Metal-containing COF as heterogeneous catalysts for PCO_2_RR

### 3.1 Pristine metallolinker COFs as photocatalyst for PCO_2_RR

Building upon the general physicochemical attributes outlined above, metallolinker-assembled COFs represent a paradigmatic platform that translates these universal design principles into structurally defined photocatalytic systems (Zhou et al., 2026). A principal distinction of this class of materials is that the coordination environment is predetermined at the monomer stage and faithfully retained during framework formation. This strategy embeds the molecular precision of homogeneous catalysts within a crystalline, porous scaffold while conferring the site isolation, substrate accessibility, and recoverability characteristic of heterogeneous materials. By judiciously selecting the ligand scaffold, metal ion, and peripheral chemical environment prior to COF assembly, the electronic structure and local coordination geometry of the catalytic center can be controlled with atomic-level precision and subsequently immobilized within an extended framework (Wang L. et al., 2024). Such covalent confinement mitigates ligand dissociation, metal leaching, and aggregation, which are common deactivation pathways in homogeneous molecular complexes (Liu et al., 2016).

#### 3.1.1 Porphyrins and expanded porphyrinoids based COFs

Porphyrins are among the most widely used metallolinkers for PCO_2_RR because their rigid N_4_ macrocyclic coordination environment combines strong visible-light absorption with broad chemical and redox tunability (Sun et al., 2025). Their characteristic Soret (∼420 nm) and Q-band (∼500–650 nm) transitions enable efficient harvesting of visible light, while *meso-*positions, together with axial coordination at the metal center, provide multiple routes for modulating π-conjugation, D–A character and metal-centered electronic states. e^−^-rich *meso*-aryl substituents can alter the energies and intensities of the Q-band transitions and promote electronic delocalization (Di Carlo et al., 2019), whereas sterically demanding substituents can modify interlayer packing, increase the accessibility of otherwise buried M–N_4_ sites and facilitate substrate transport. In metallolinker-assembled porphyrinic COFs, metalation is commonly established before framework synthesis, allowing the desired metal–N_4_ coordination environment to be incorporated directly into the crystalline lattice and promoting uniform spatial distribution of the catalytic sites.

Lu *et al.* illustrated the influence of the central metal on framework photophysics by preparing a series of crystalline **TTCOF-M** materials (M = 2H, Zn, Ni and Cu) using tetrathiafulvalene (**TTF**) as an e^−^-donating antenna and metalloporphyrins as photoactive acceptors and catalytic nodes (Figure 2a). Systematic variation of the porphyrin metal center produced substantial differences in optical and excited-state behavior. Among the series, **TTCOF-Zn** exhibited the narrowest band gap (1.49 eV), the strongest photoluminescence quenching and the longest fluorescence lifetime, consistent with more favorable charge-separation dynamics. The authors attributed this behavior to the *d* ^10^ configuration of Zn^II^, which limits ligand-to-metal charge-transfer (LMCT) pathways and modifies excited-state relaxation, thereby increasing the availability of photogenerated electrons for catalytic reduction. **TTCOF-Zn** consequently produced 12.33 μmol of CO with approximately 100% selectivity while simultaneously oxidizing H_2_O to O_2_, demonstrating CO_2_ reduction without an added SED. Mechanistically, visible-light excitation promotes photoinduced electron transfer from the **TTF** donor to the metalloporphyrin acceptor, directing reducing equivalents toward the metallolinker for CO_2_ conversion while retaining oxidative equivalents on the **TTF** units for H_2_O oxidation. This comparison shows that changing only the central metal within an otherwise related framework can substantially alter excited-state dynamics and photocatalytic behavior, highlighting the sensitivity of metallolinker-COFs to metal-dependent electronic structure (Lu et al., 2019).

**FIGURE 2:**
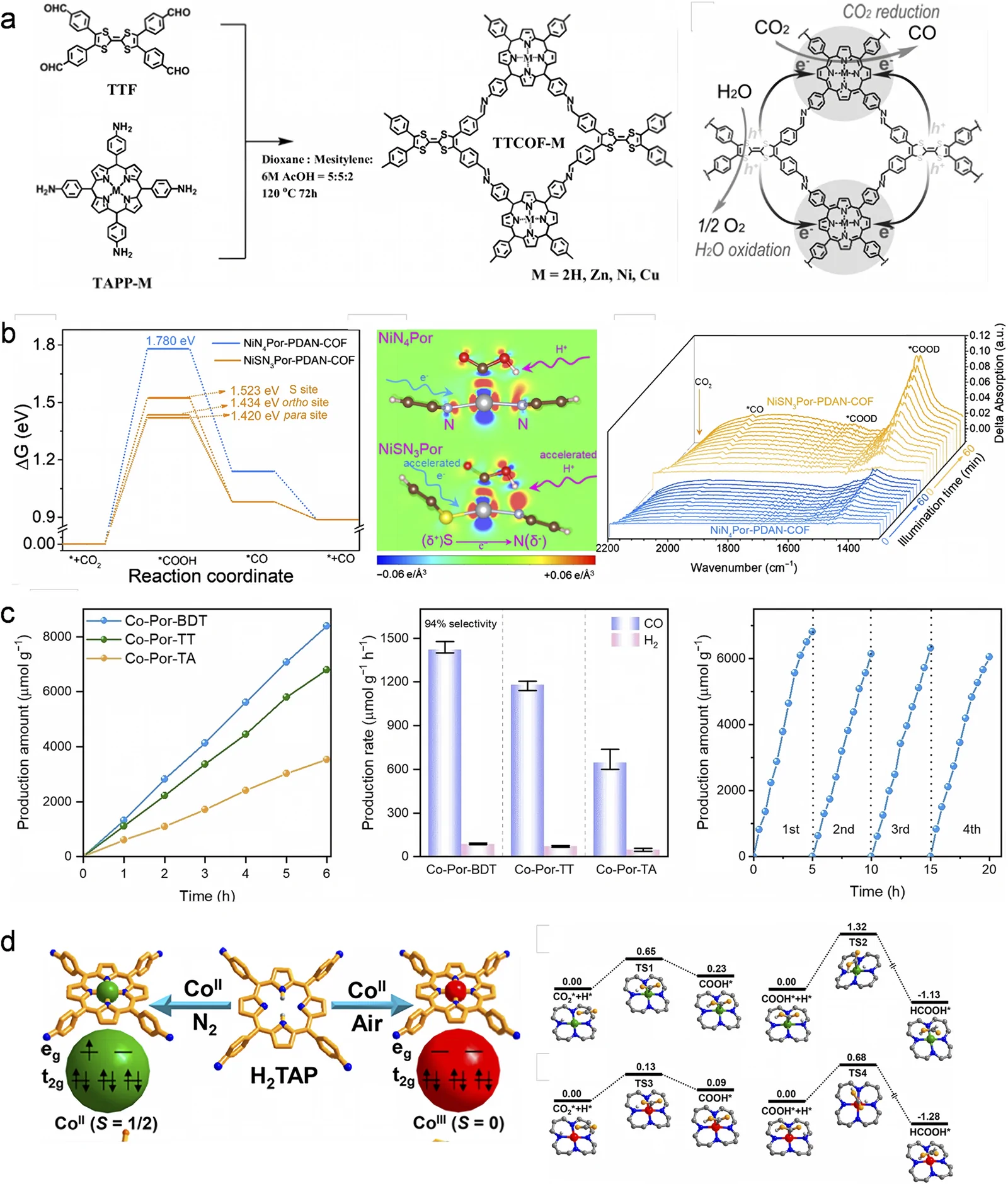
**(a)** Schematic of the synthesis of **TTCOF-M** and the mechanism of CO_2_RR with H_2_O oxidation. Reproduced with permission (Lu et al., 2019). Copyright 2019, Wiley-VCH GmbH. **(b)** Gibbs free energy diagrams of CO_2_ conversion pathways, the differential charge density of COOH with **NiN**_**4**_ **Por** and **NiSN**_**3**_ **Por** fragments, and the change of *in situ* FT-IR spectra of **NiSN**_**3**_ **Por-PDAN-COF** and **NiN**_**4**_ **Por-PDAN-COF**. Reproduced with permission (Wang et al., 2024c). Copyright 2025, Chinese Chemical Society. **(c)** Time-dependent CO production yield, CO production rate and selectivity, long-term stability test results of **Co-Por-BDT**. Reproduced with permission (Kim YH. et al., 2023). Copyright 2023, Wiley-VCH GmbH. **(d)** Fabrication of **COF-367-Co** featuring different spin states of Co ions, calculated potential energy profile of CO_2_ reduction reaction to HCOOH. Reproduced with permission (Gong et al., 2020). Copyright 2020, American Chemical Society.

Beyond the choice of central metal, the symmetry of the porphyrinic metallolinker represents an additional molecular-design variable that can be precisely programmed at the monomer stage and retained within the crystalline COF lattice. Wang *et al.* demonstrated this principle through a desymmetrization strategy, replacing one pyrrole ring in a Ni-porphyrin monomer with thiophene to afford **NiSN**_**3**_ **Por**, which was subsequently condensed with *p*-phenylenediacetonitrile (**PDAN**) to yield **NiSN**_**3**_ **Por-PDAN-COF** (Figure 2b). X-ray absorption spectroscopy confirmed that introducing sulfur breaks the *D*_4h_ symmetry of the Ni coordination environment and increases the local charge density around the Ni center, while the compression of the Ni–N bond length from 1.93 to 1.82 Å further evidences the structural distortion imposed by the bulkier thiophene subunit. Remarkably, this symmetry-breaking design enables a “divide-and-conquer” functional regionalization within a single crystalline framework: femtosecond transient absorption spectroscopy revealed that the thiophene moiety acts as a high-speed e^−^-transport channel, accelerating both LMCT and MLCT events relative to the symmetric **NiN**_**4**_ **Por** counterpart; theoretical analysis and FT-IR studies further established that the remaining pyrrole rings serve as proton-hopping sites, while the Ni center functions as the CO_2_-binding site. As a direct consequence of this spatially separated charge- and mass-transport engineering, **NiSN**_**3**_ **Por-PDAN-COF** achieves a CO production rate of 22.7 mmol·g^−1^·h^−1^ under visible light (λ > 420 nm), representing a ∼65-fold enhancement over its *D*_4h_-symmetric analog **NiN**_**4**_ **Por-PDAN-COF**. This study exemplifies how the molecular precision afforded by pre-metalated monomers can be exploited to transcend the limitations of conventional symmetric porphyrinic COFs, wherein the isotropic distribution of e^−^- and proton-transport pathways constrains overall photosynthetic efficiency (Wang et al., 2024c).

The framework surrounding a metalloporphyrin is equally important because the catalytic properties of the metal center are coupled to the electronic structure of the extended π-system. Kim *et al.* established this relationship using a D–A series in which an e^−^-deficient Co-porphyrin (**Co-Por**) served as the invariant tetravalent acceptor and catalytic node, while benzo[1,2-*b*;4,5-*b*]dithiophene (**BDT**), thieno[3,2-*b*]thiophene (**TT**) and terephthaldehyde (**TA**) were introduced as dicarbaldehyde donors with progressively different π-conjugation lengths, yielding **Co-Por-BDT**, **Co-Por-TT** and **Co-Por-TA**, respectively (Figure 2c). Increasing donor conjugation in the order **BDT** > **TT** > **TA** enhanced e^−^ delocalization and D-to-A electronic coupling, reducing the exciton binding energy from 0.16 eV in **Co-Por-TA** and 0.14 eV in **Co-Por-TT** to 0.10 eV in **Co-Por-BDT**. The optical band gap decreased in parallel, from 1.94 eV for **Co-Por-TA** and 1.87 eV for **Co-Por-TT** to 1.80 eV for **Co-Por-BDT**. These changes were accompanied by progressively longer photogenerated charge lifetimes of 0.92, 1.55 and 2.15 ns, respectively, indicating more effective charge separation as framework conjugation increased. Photocatalytic CO_2_-to-CO activity followed the same trend, with evolution rates of 642 μmol·g^−1^·h^−1^ for **Co-Por-TA**, 1,182 μmol·g^−1^·h^−1^ for **Co-Por-TT** and 1,424 μmol·g^−1^·h^−1^ for **Co-Por-BDT**. All three COFs maintained approximately 94% selectivity for CO and showed only marginal activity loss over four recycling runs. These results demonstrate that the metallolinker alone does not determine photocatalytic performance; rather, its interaction with the surrounding conjugated framework governs exciton binding, charge separation and carrier transport, which ultimately determine the efficiency of e^−^ delivery to the catalytic Co center (Kim Y.H. et al., 2023).

Metal oxidation and spin states provide further control over intermediate binding and product selectivity. Gong *et al.* examined this effect in **COF-367-Co** by varying the electronic state of the cobalt center while retaining the same porphyrinic framework (Figure 2d). **Co** ^**II**^ **-TAP** synthesized under N_2_ and **Co** ^**III**^ **-TAP** obtained through controlled oxidation in air generated Co^II^ and Co^III^ centers with reported spin states of *S* = 1/2 and *S* = 0, respectively. The change in cobalt electronic structure was supported by the disappearance of the *g* = 2.30 EPR signal for Co^III^, a shift of the X-ray absorption near-edge structure (XANES) absorption edge to higher energy, and a decrease in the Co 2*p* spin–orbit splitting from 16 eV for Co^II^ to 15 eV for Co^III^. These changes produced a marked shift in PCO_2_RR selectivity. **COF-367-Co** ^**III**^ generated HCOOH at 93.0 ± 4.63 μmol·g^−1^·h^−1^, together with 5.5 μmol·g^−1^·h^−1^ of CO and 10.1 μmol·g^−1^·h^−1^ of CH_4_, whereas **COF-367-Co** ^**II**^ produced substantially less HCOOH (48.6 μmol·g^−1^·h^−1^) but higher amounts of CO (16.5 μmol·g^−1^·h^−1^) and CH_4_ (12.8 μmol·g^−1^·h^−1^). DFT calculations attributed this divergence to oxidation-state-dependent orbital interactions with adsorbed CO_2_. In **COF-367-Co** ^**II**^, the O 2*p* orbitals of adsorbed CO_2_ interact predominantly with Co 3*d*_xz_/3*d*_yz_ states, whereas in **COF-367-Co** ^**III**^ stronger coupling with the Co 3*d*_*z*_^2^ orbital increases the calculated Co–O adsorption energy from 0.05 to 0.11 eV and shortens the Co–O distance from 2.82 to 2.31 Å. This altered coordination lowers the calculated rate-determining barrier for HCOOH formation from 1.32 eV on Co^II^ to 0.68 eV on Co^III^, while increasing the barrier for subsequent HCOOH conversion toward CO or CH_4_ from 0.97 to 1.20 eV. This selectivity shift shows how changes in the oxidation and spin state of a structurally confined metallolinker can reorganize metal–adsorbate orbital interactions and reshape the free-energy landscape of competing CO_2_ reduction pathways (Gong et al., 2020).

#### 3.1.2 Phthalocyanines based COFs

Phthalocyanines (**Pc**) are structurally related to porphyrins but possess a more extensively π-conjugated macrocyclic framework, giving rise to intense absorption at longer wavelengths, typically with Q-bands in the ∼650–750 nm region. This extended conjugation makes metallophthalocyanines attractive light-harvesting and catalytic units for PCO_2_RR, although their limited solubility and more demanding synthesis and processing can complicate framework construction (Liu et al., 2026). Within COFs, however, these limitations can be partially mitigated by covalent integration of the phthalocyanine macrocycle into an ordered scaffold, where its strong visible-to-red-light absorption, M–N_4_ coordination environment and extended π-system can be coupled directly to framework-mediated charge transport and substrate activation.

Lin *et al.* demonstrated the importance of the local coordination environment using a series of dioxin-linked **EPM-COFs** (M = Co, Ni and Cu) constructed from perfluorinated metallophthalocyanines (**MPcF**_**16**_) and naturally derived ellagic acid (**EA**) as the e^−^-donating organic component (Figure 3). Among these materials, **EPCo-COF** exhibited a CO evolution rate of 14.1 mmol·g^−1^·h^−1^ with 85.1% selectivity, consistent with the Co-N_4_ phthalocyanine units functioning as well-defined catalytic centers. More importantly, partial alkaline hydrolysis of ester-containing groups within the **EA** component generated additional carboxylate (COO^−^) and hydroxyl (OH) functionalities without substantially disrupting the crystalline framework or altering the primary Co–N_4_ coordination motif, yielding **EPCo-COF-AT**. This post-synthetic modification of the surrounding chemical environment increased the e^−^-donating character of the **EA**-derived framework, shifted the CB edge to a more reducing potential and promoted intraframework e^−^ transfer toward the **CoPcF**_**16**_ metallolinker. Femtosecond transient-absorption measurements showed a decrease in the average kinetic time constant (τ_avg_) from 107.5 to 72.2 ps, consistent with accelerated excited-state charge-transfer dynamics; electrochemical impedance measurements and DFT calculations further supported more efficient electronic communication between the framework and the **CoPc** unit. The calculated barrier for the RDS decreased from 0.82 to 0.43 eV after alkaline treatment. Correspondingly, **EPCo-COF-AT** achieved a CO production rate of 17.7 mmol·g^−1^·h^−1^ with 97.8% selectivity, a substantial improvement over the untreated framework and comparable to visible-light-driven COF photocatalysts reported at the time. These results illustrate an important design principle for metallolinker-COFs: catalytic performance can be regulated not only through the metal center’s primary coordination sphere but also through chemical modification of the surrounding framework, which controls charge delivery and intermediate energetics without replacing the catalytic metal site (Lin et al., 2024).

**FIGURE 3:**
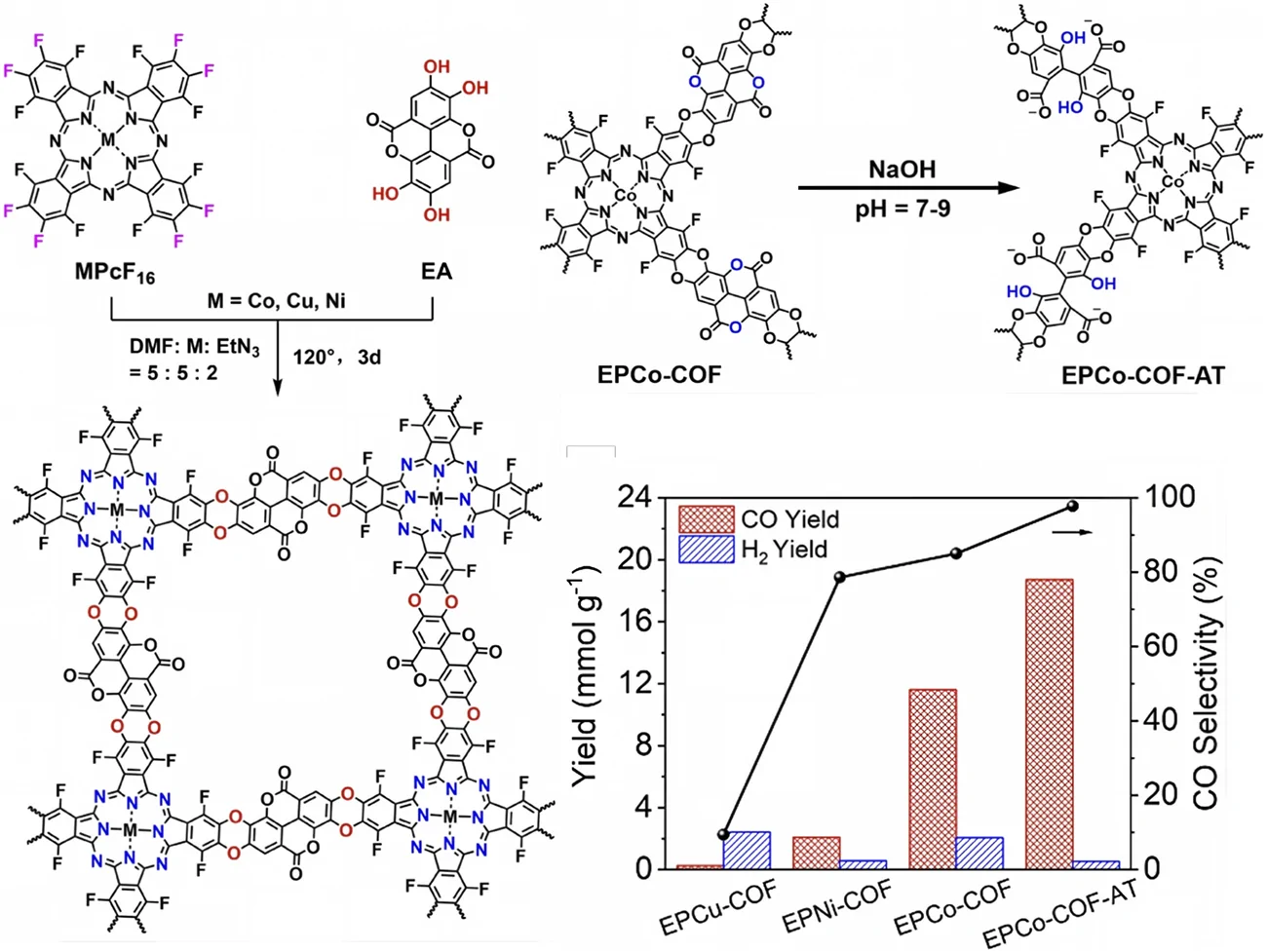
Schematic synthesis of **EPM-COF** (M = Co, Cu, Ni), comparison of the photocatalytic activity of the **EPM-COF**. Reproduced with permission (Lin et al., 2024). Copyright 2024, American Chemical Society.

The same group subsequently used morpholine-linked Co–phthalocyanine COFs to isolate the effect of framework π-conjugation on charge transport and photocatalytic activity. **Co-DA-COF** and **Co-DB-COF** were synthesized through nucleophilic aromatic substitution of cobalt perfluorophthalocyanine (**CoPcF**_**16**_) with 2,5-phenylenediamine-1,4-diphenol (**DHA**) and 3,3′-dihydroxybenzidine (**DHB**), respectively. Both materials adopted AB-stacked tetragonal structures assigned to the *I*4/*m* space group, with Pawley refinement giving *R*_p_ values of 3.14% for **Co-DA-COF** and 2.90% for **Co-DB-COF**. Characteristic C–O–C stretching bands at 1,284 and 1,145 cm^−1^ in the FT-IR spectra, together with solid-state ^13^C NMR analysis, supported formation of the morpholine-type linkages, indicating that modification of the organic linker could be achieved while retaining the crystalline metallophthalocyanine framework. The principal difference between the two COFs was the extent of π-conjugation in the donor component: the biphenyl-like **DHB** unit generated a more extended conjugated pathway in **Co-DB-COF** than the shorter **DHA**-derived unit in **Co-DA-COF**. This structural difference was reflected in their photophysical properties. **Co-DB-COF** displayed weaker photoluminescence, a longer average exciton lifetime (τ_avg_ = 2.93 ns *vs*. 1.90 ns for **Co-DA-COF**), a stronger photocurrent response and a smaller electrochemical impedance arc, collectively indicating more effective separation and transport of photogenerated charge carriers. DFT calculations further showed that the LUMO of both frameworks is concentrated predominantly on the **CoPcF**_**16**_ metallolinker, with a substantial contribution from the Co 3*d*_xz_ orbital, whereas the HOMO is mainly localized on the organic donor. Notably, the calculated LUMO of **Co-DB-COF** (−3.711 eV) is higher in energy, that is, less negative, than that of **Co-DA-COF** (−3.775 eV), consistent with altered reducing energetics following extension of the conjugated framework. The calculations also yielded reported *COOH formation energies of −0.13 eV for **Co-DB-COF** and −0.29 eV for **Co-DA-COF**; the authors associated the modified energetic landscape with an approximately 0.16 eV decrease in the kinetic barrier for the initial hydrogenation step. Under visible-light irradiation (λ ≥ 420 nm), using [Ru(bpy)_3_]Cl_2_ as the photosensitizer and TEOA as the SED, **Co-DB-COF** produced CO at 25.7 mmol·g^−1^·h^−1^ with 92.3% selectivity and an apparent quantum efficiency of 0.65% at 450 nm. The framework also retained its crystallinity, elemental composition and photocatalytic activity over at least five recycling cycles. Taken together, these results show that the metallophthalocyanine center and the surrounding π-conjugated framework operate as electronically coupled components: extending the donor conjugation alters Frontier-orbital energetics and charge-carrier dynamics, thereby regulating the efficiency with which photogenerated − are delivered to the **CoPc** catalytic site (Lin et al., 2025).

#### 3.1.3 Salen and salophen based COFs

Salen and salophen ligands provide non-macrocyclic N_2_O_2_ coordination pockets whose metal identity, ligand substitution and axial coordination can be varied more readily than in many porphyrins or phthalocyanine systems. Their incorporation into COFs therefore provides a useful platform for separating the effects of metal-site chemistry from those of framework formation and morphology.

Zhou *et al.* developed a scalable one-step multicomponent synthesis of metallosalen COFs by combining the salen-forming aldehyde precursor, metal salt and amine monomer within the same reaction mixture (Figure 4). This strategy afforded mononuclear **Zn-TAPB-COF-1** and **Co-TAPT-COF-1** as well as the binuclear **ZnZn-TAPB-COF-1**, containing well-defined M–N_2_O_2_ and M–N_2_O_2_–M–N_2_O_2_ coordination motifs supported by XAFS, AC-TEM and EXAFS. Importantly, the synthetic route affected more than metal incorporation. The simultaneous presence of all building components preserved reversible imine exchange during framework formation, facilitating error correction and yielding substantially higher crystallinity than the corresponding two-step metalation route. Consequently, **Co-TAPT-COF-1** obtained by the one-step route assembled into hollow nanotubes with a macroporous central channel, whereas **Co-TAPT-COF-2** prepared by the stepwise route formed irregular nanosheets and exhibited lower crystallinity and partial pore collapse. These structural differences translated directly into photocatalytic behavior. **Zn-TAPB-COF-1** and **ZnZn-TAPB-COF-1** predominantly favored H_2_ evolution, whereas Co incorporation increased CO formation and enabled syngas production. **Co-TAPB-COF-1** generated H_2_ and CO at 6.71 and 6.44 mmol·g^−1^·h^−1^, respectively, while nanotubular **Co-TAPT-COF-1** reached 11.31 mmol·g^−1^·h^−1^ H_2_ and 8.39 mmol·g^−1^·h^−1^ CO, corresponding to a total syngas production rate of 19.7 mmol·g^−1^·h^−1^. By comparison, structurally analogous **Co-TAPT-COF-2** produced only 4.72 mmol·g^−1^·h^−1^ H_2_ and 3.50 mmol·g^−1^·h^−1^ CO. DFT calculations identified formation of *COOH as the RDS for CO production and gave a free-energy requirement of 0.75 eV for **Co-TAPT-COF**, substantially below those calculated for the corresponding Zn- and ZnZn-containing models. Thus, the activity difference between the two **Co-TAPT** materials cannot be attributed simply to metal identity or bulk band-gap energetics, which are similar, but instead reflects the consequences of crystallinity, pore integrity and nanotubular morphology for substrate transport and photogenerated-carrier migration. This comparison is particularly instructive because it shows that the synthetic pathway itself can become a functional design variable: the same nominal coordination chemistry can produce markedly different catalytic behavior when framework growth and mesoscale organization are altered (Zhou et al., 2023).

**FIGURE 4:**
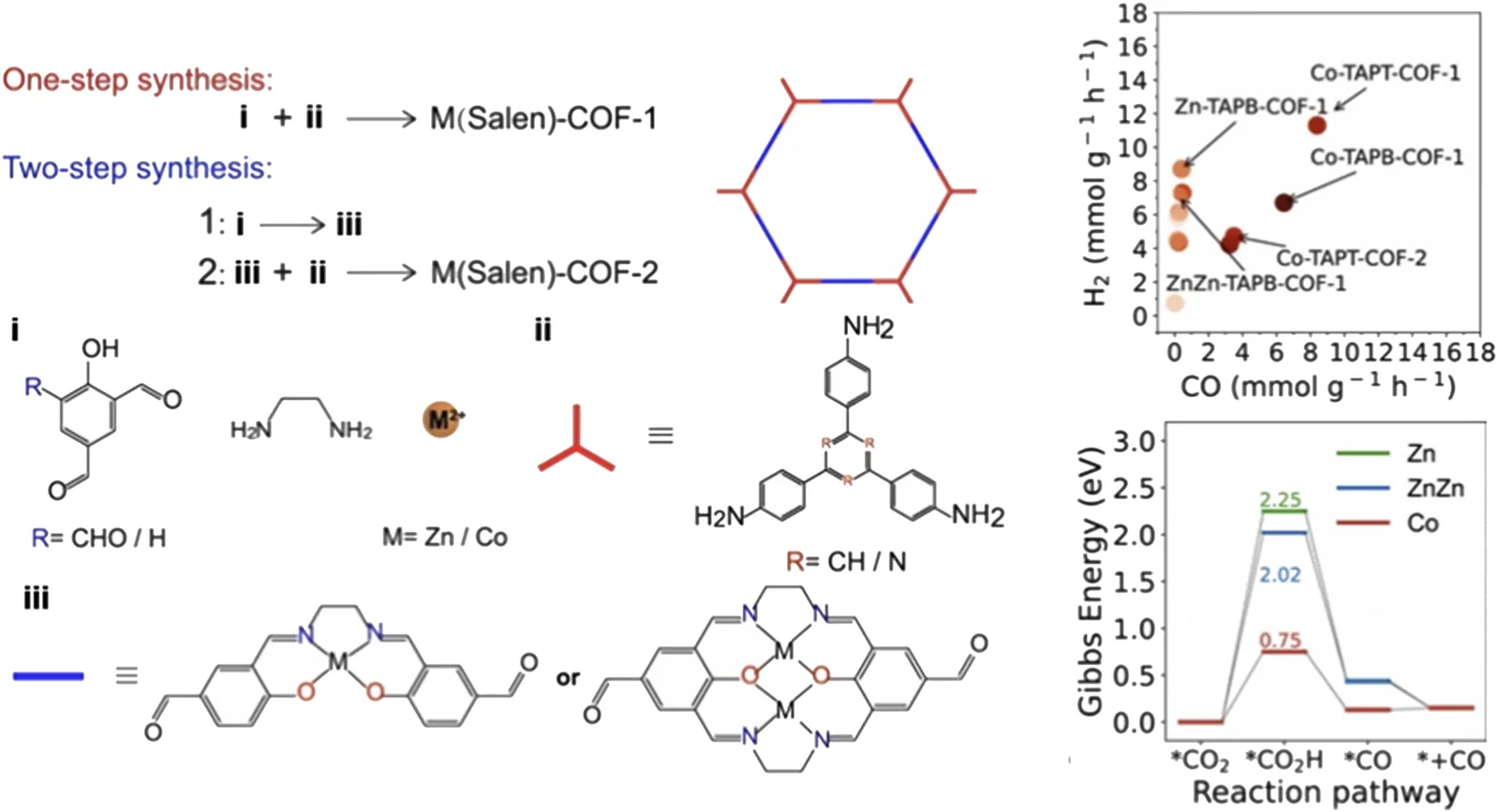
Synthesis strategies of metallosalen-covalent organic frameworks (M(salen)-COFs) (M = Zn and Co), syngas production rate with M(salen), calculated free energy diagrams for CO_2_ reduction to CO on **Zn-TAPT-COF**, **ZnZn-TAPT-COF** and **Co-TAPT-COF**. Reproduced with permission (Zhou et al., 2023). Copyright 2023, Springer Nature.

The catalytic consequences of salen-site organization become still more apparent when two neighboring metal centers are deliberately incorporated into the same coordination motif. Dong, Zhang, Lan *et al.* developed dual-metallosalen COFs (**MM-Salen-COF**; M = Zn, Ni and Cu) through one-step acid-catalyzed Schiff-base condensation of (1*R*,2*R*)-(−)-1,2-diaminocyclohexane with 5,5′,5″-(1,3,5-triazine-2,4,6-triyl)tris(2-hydroxyisophthalaldehyde) in the presence of M(OAc)_2_·*n*H_2_O, and compared them with single-metal **M-Salen-COFs** derived from 1,3,5-triazine-2,4,6-tris(4′-hydroxy-5′-formylphenyl)benzene. For **ZnZn-Salen-COF**, Pawley-refined PXRD (*R*_wp_ = 5.24%) supported a staggered AA-stacked *P*321 structure with an interlayer separation of 3.34 Å, while EXAFS and wavelet-transform analysis indicated Zn–N_2_O_2_ coordination with Zn–N and Zn–O distances of approximately 1.94 and 2.03 Å, respectively. The material contained 13.07 wt% Zn without a detectable Zn–Zn scattering contribution, consistent with neighboring but non-metallically bonded Zn sites embedded within the salen framework. Under visible light (420–800 nm) and without an additional cocatalyst or molecular photosensitizer, **ZnZn-Salen-COF** produced CO at 150.9 μmol·g^−1^·h^−1^ under pure CO_2_, approximately sixfold higher than the 25.5 μmol·g^−1^·h^−1^ obtained with **Zn-Salen-COF**. More significantly, at 15% CO_2_ in N_2_, used to approximate the CO_2_ concentration of flue gas, the dual-site framework retained a CO evolution rate of 102.1 μmol·g^−1^·h^−1^, whereas its single-Zn analog produced only 14.4 μmol·g^−1^·h^−1^. *In situ* diffuse reflectance infrared Fourier transform spectroscopy (DRIFTS) detected the *COOH intermediate for **ZnZn-Salen-COF** but not for **Zn-Salen-COF**, linking the activity enhancement to more effective activation of dilute CO_2_ at adjacent Zn sites. DFT calculations supported a cooperative adsorption mechanism in which the two neighboring centers stabilize the adsorbed intermediate and increase charge transfer to *COOH; the associated Bader-charge analysis gave values of approximately 0.40 *e* and 0.24 *e* for the dual- and single-site models, respectively. Correspondingly, the calculated free-energy requirement for the rate-determining CO_2_-reduction step decreased from 2.00 eV on the single-Zn site to 0.69 eV on the dual-Zn motif. The binuclear environment also influenced the oxidative half-reaction, lowering the calculated energetic requirement for *OOH formation from 2.36 to 1.94 eV. The mechanistic significance therefore extends beyond a simple increase in metal-site density: positioning two coordination centers at a defined separation creates a cooperative adsorption geometry that changes the electronic stabilization of key intermediates and preserves activity when CO_2_ availability becomes limiting. This transition from isolated to cooperatively coupled coordination sites illustrates how molecular concepts derived from dinuclear complexes can be encoded periodically within a porous photocatalytic lattice to address reaction regimes, such as direct conversion of dilute CO_2_, that remain difficult for conventional single-site COFs (Dong et al., 2025).

### 3.2 Post-synthetically metalated COFs for PCO_2_RR

#### 3.2.1 Bipyridine and imine-pyridine scaffolds

Bipyridine and related N-donor motifs provide well-defined chelation environments that can be incorporated periodically into COFs and subsequently coordinated with catalytically active metal ions. Unlike the pre-metalated porphyrin and **Pc** systems discussed above, many bipyridine-containing COFs are metalated post-synthetically. They are therefore treated here as post-synthetically metalated COFs rather than as metallolinker COFs, but they remain mechanistically informative because they allow the coordination environment, framework electronics and pore architecture to vary independently. Together, these systems illustrate how metal-site identity, secondary coordination environment, linkage chemistry, interlayer packing and D–A coupling regulate CO_2_ activation and photogenerated charge transfer.

Although it is neither a bipyridine-based metallolinker COF nor a photocatalyst for CO_2_ reduction, the **C**_**2**_ **O** framework reported by Kim *et al.* provides a useful conceptual comparison for framework-controlled catalytic microenvironments. **C**_**2**_ **O** is a two-dimensional (2D) crystalline framework obtained by self-condensation of trichlorophloroglucinol through irreversible nucleophilic aromatic substitution, generating chemically robust dioxin linkages and allowing gram-scale synthesis. The framework remains stable under acidic, basic and organic-solvent conditions and contains rigid crown-ether-like pores that coordinate K^+^. In the KI-cocatalyzed cycloaddition of CO_2_ with epoxides, K^+^ binding within these pores enhances the nucleophilicity of I^−^ and substantially lowers the calculated barrier for the rate-determining epoxide ring-opening step from 63.96 to 28.01 kcal·mol^−1^. Correspondingly, CO_2_ conversion increased from 5.7% to 99.9% for epichlorohydrin and from 2.9% to 74.2% for allyl glycidyl ether. **C**_**2**_ **O** also contains electrophilic–Cl and nucleophilic–OH groups at its edges, allowing post-synthetic modification with allyl glycidyl ether or ethoxyethylamine to tune interactions between the framework and reaction medium; these modifications increased allyl glycidyl ether conversion to 97.2% and 99.9%, respectively. Although this chemistry concerns thermal CO_2_ fixation rather than PCO_2_RR, it demonstrates that a rigid porous lattice can organize ion-binding sites and peripheral functionalities to reshape the local reaction environment and lower elementary-step barriers (Kim et al., 2024).

A more direct example is Zhang *et al.* constructed a 2D imine-linked **Bpy-COF** containing periodically arranged bipyridine chelation sites for post-synthetic metalation (Figure 5a). Among the resulting **M-Bpy-COF**, **Cu-Bpy-COF** contains atomically dispersed Cu–N_x_ sites, as supported by Extended X-ray absorption fine structure (EXAFS), and exhibits an optical band gap of 2.36 eV. Incorporation of Cu redistributes the Frontier electronic states toward the Cu–bipyridine unit and promotes localization of photogenerated e^−^ at the catalytic center, thereby suppressing charge recombination. Under visible-light irradiation, **Cu-Bpy-COF** produced 70.0 μmol g^−1^ CH_4_ within 4 h in CO_2_-saturated aqueous medium containing triethylamine (TEA), without an additional molecular photosensitizer. Importantly, changing the reaction medium altered the product distribution without modifying the metal center, ligand or framework topology: in the aqueous proton-rich environment, TEA-assisted PCET favored deeper reduction to CH_4_, whereas in DMF, where proton transfer was strongly restricted, CO became the dominant product with >90% selectivity. This solvent-dependent switch highlights the importance of the reaction microenvironment in determining whether adsorbed carbon intermediates undergo further protonation or desorb after two-electron reduction. Enhanced CO_2_ uptake, improved wettability, and recyclability further support the porous framework’s ability to couple substrate accessibility with a structurally defined Cu coordination site. The study therefore shows that product selectivity in metalated bipyridine COFs is governed not only by the primary Cu–N coordination sphere but also by proton availability and the surrounding solvent environment (Zhang Y. et al., 2023).

**FIGURE 5:**
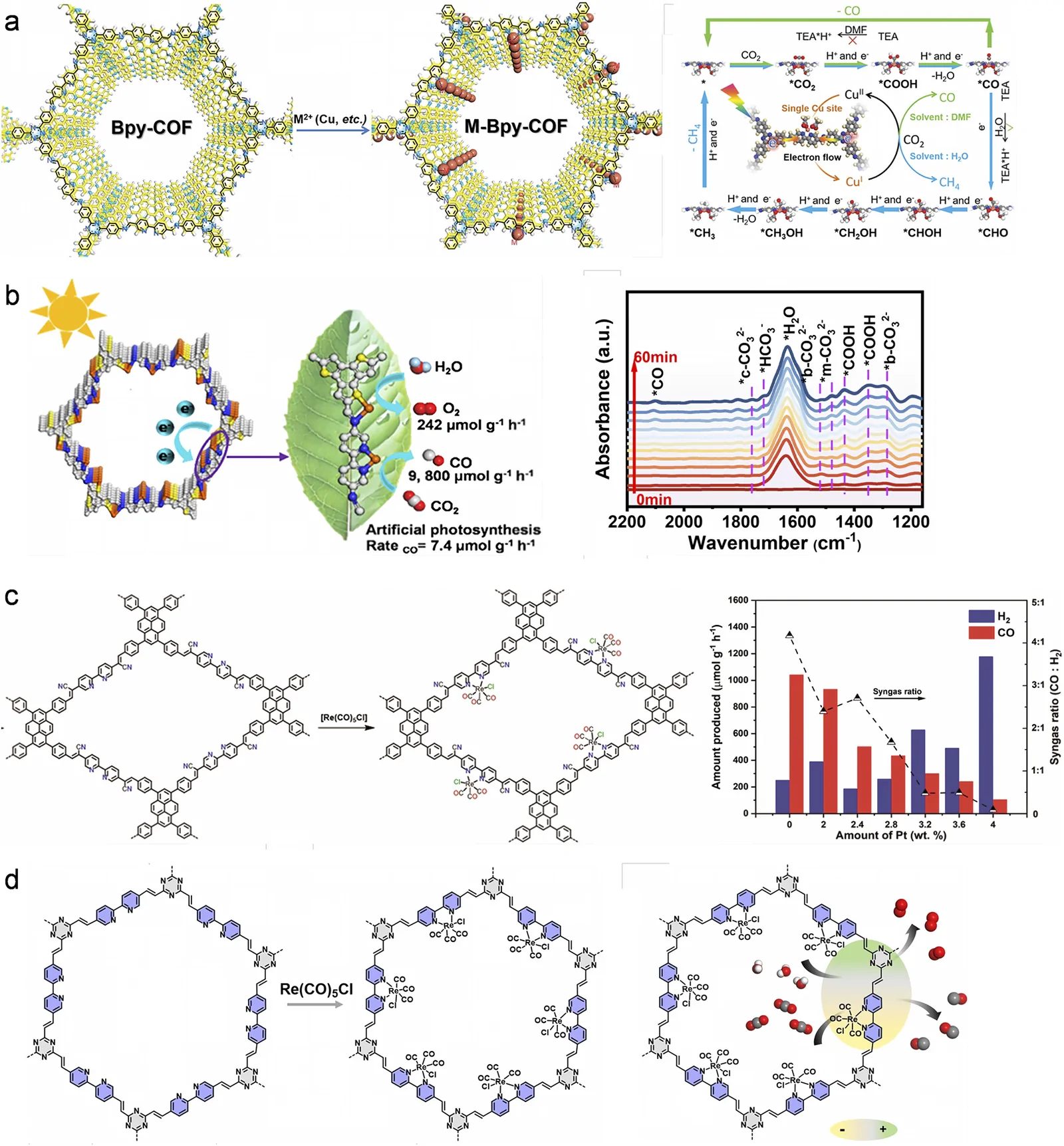
**(a)** Synthetic scheme of **Bpy-COF** and its postsynthetic modification to **M-Bpy-COF**, proposed reaction mechanism for photocatalytic CO_2_-to-CO in DMF or CH_4_ in H_2_O conversion over **Cu-Bpy-COF**. Reproduced with permission (Zhang Y. et al., 2023). Copyright 2023, Wiley-VCH GmbH. **(b)** Schematic of the **Btt-Bpy-COF** in photocatalytic CO_2_RR, *in situ* ATR FT-IR spectra of CO_2_ and H_2_O interactions with **Co-Btt-Bpy-COF** under subsequent light irradiation. Reproduced with permission (Xu et al., 2025). Copyright 2025, American Chemical Society. **(c)** Synthesis of **Bpy-sp** ^**2**^ **c-COF** and **Re-Bpy-sp** ^**2**^ **c-COF**, photocatalytic syngas generation of Pt modified **Re-Bpy-sp** ^**2**^ **c-COF** under visible light irradiation. Reproduced with permission (Fu et al., 2020). Copyright 2020, the Royal Society of Chemistry. **(d)** The synthesis route to **viCOF-bpy** and **viCOF-bpy-Re**, the mechanism of photocatalytic CO_2_ reduction by using **viCOF-bpy-Re** as photocatalyst. Reproduced with permission (Cheng et al., 2023). Copyright 2023, Wiley-VCH GmbH.

The coordination environment can also be diversified within a single framework to couple the reduction and oxidation half-reactions. Xu *et al.* reported the D–A **Btt-Bpy COF** containing two distinct Co coordination motifs: an N–Co–N environment associated with bipyridine units for CO_2_ reduction and an N–Co–S environment within the thiophene–imine domain for H_2_O oxidation (Figure 5b). The intrinsic D–A architecture promotes intraframework charge transfer and spatial separation of photogenerated carriers, allowing the two coordination environments to participate preferentially in different half-reactions. **Co-Btt-Bpy** produced CO at 9,800 μmol·g^−1^·h^−1^ and O_2_ at 242 μmol·g^−1^·h^−1^ under visible-light photocatalytic conditions and, under sacrificial-agent-free conditions, sustained CO evolution at 7.4 μmol·g^−1^·h^−1^. *In situ* attenuated total reflectance Fourier-transform infrared spectroscopy (ATR-FT-IR) measurements combined with DFT calculations attributed this bifunctional behavior to complementary roles of the electrophilic N–Co–N and nucleophilic N–Co–S environments, which lower the energetic requirements for forming key *COOH and *OH intermediates, respectively. At the same time, spatial organization of the two sites within the D–A framework facilitates charge separation and directs reducing and oxidizing equivalents toward their corresponding catalytic centers. This system shows how one crystalline architecture can encode chemically distinct coordination motifs to couple CO_2_ reduction with H_2_O oxidation, rather than treating the two half-reactions as independent catalytic processes (Xu et al., 2025).

Framework geometry provides an additional level of regulation even when chemical composition is unchanged. Song *et al.* compared two pyrene–bipyridine COFs generated by condensation of 2,2′-bipyridine-5,5′-diamine with the geometrical tetra-aldehyde isomers **K-Py-4CHO** and **A-Py-4CHO**, yielding **K-Py-Bpy-COF** and **A-Py-Bpy-COF**, respectively. Single-crystal analysis of the aldehyde precursors showed a substantially larger torsion angle between the formylphenyl substituent and pyrene core in **K-Py-4CHO** (86.2°) than in **A-Py-4CHO** (56.2°). This difference propagates into the assembled frameworks, increasing the interlayer separation from 3.76 Å in the A-isomer to 4.39 Å in the K-isomer and weakening interlayer π–π interactions. Consequently, the two COFs exhibit distinct excited-state properties, with optical band gaps of 2.64 and 2.28 eV for the K- and A-derived frameworks, respectively; notably, 2.64 eV is larger rather than narrower, so the enhanced activity of the K-isomer cannot be attributed simply to band-gap narrowing. Instead, the K-framework exhibits more favorable intralayer charge-transfer behavior and reduced excited-state quenching. Following post-synthetic incorporation of Co, XANES, EXAFS and aberration-corrected high-angle annular dark-field scanning transmission electron microscopy (AC-HAADF-STEM) supported atomically dispersed Co^2+^ centers in Co–N coordination environments without detectable Co–Co scattering. Under visible-light irradiation with [Ru(bpy)_3_]Cl_2_ as the photosensitizer and TEOA as the sacrificial donor, **K-Py-Bpy-COF-Co-75%** produced CO at 12,476.4 μmol·g^−1^·h^−1^ with 93.3% selectivity, compared with 2,174.4 μmol·g^−1^·h^−1^ for **A-Py-Bpy-COF-Co**, corresponding to a 5.7-fold difference. The K-isomer also exhibited a longer excited-state lifetime (1.80 *vs*. 0.77 ns), a larger Co 2*p* spin–orbit splitting (15.52 eV), and a lower calculated *COOH formation barrier (0.38 *vs*. 0.81 eV). These results indicate that subtle changes in precursor geometry can propagate across structural length scales, from torsional configuration to interlayer separation and excited-state dynamics, and ultimately alter the electronic environment and catalytic accessibility of an otherwise chemically similar Co site (Song et al., 2025).

A related strategy exploits the orientation of the covalent linkage itself to regulate interlayer electronic coupling. Qin *et al.* prepared two isomeric imine-linked COFs, **PY-CN-BIP** and **PY-NC-BIP**, from the same pyrene donor and 2,2′-bipyridine acceptor motifs but with reversed imine-bond polarity (C^δ+^ = N^δ−^ *vs*. N^δ−^ = C^δ+^). Post-synthetic coordination of Ni generated **PY-CN-BIP-Ni** and **PY-NC-BIP-Ni** with atomically dispersed Ni–bipyridine sites assigned to Ni–N_2_Cl_2_ coordination by EXAFS, XANES and AC-HAADF-STEM. Without an external photosensitizer or cocatalyst, **PY-CN-BIP-Ni** produced CO at 553.3 μmol·g^−1^·h^−1^ with 94% selectivity under visible light, compared with 73.6 μmol·g^−1^·h^−1^ for **PY-NC-BIP-Ni**. DFT calculations and molecular-dynamics simulations associated this approximately eightfold difference with linkage-dependent electronic polarization and interlayer coupling. In **PY-CN-BIP**, connecting pyrene to the e^−^-deficient imine carbon modifies the donor-state energetics, whereas placing the imine nitrogen adjacent to bipyridine alters the acceptor states, generating an asymmetric charge distribution that weakens interlayer π–π interactions and increases electronic confinement within individual layers. Femtosecond transient absorption, temperature-dependent photoluminescence and EPR measurements were consistent with more efficient intralayer charge transfer from pyrene toward the Ni–bipyridine catalytic sites. By contrast, stronger interlayer coupling in **PY-NC-BIP** promotes excimer formation between pyrene units and increases charge trapping at the D–A interface. Exfoliation provided an additional test of this interpretation: conversion of the bulk materials into ultrathin nanosheets increased the photocatalytic activity of **PY-NC-BIP-Ni** by 6.4-fold but that of **PY-CN-BIP-Ni** by only 2.4-fold, consistent with interlayer interactions imposing a larger kinetic penalty in the former. This comparison demonstrates that linkage orientation can regulate photocatalysis indirectly by controlling interlayer electronic communication rather than simply altering the local coordination structure of the metal center (Qin et al., 2025).

Linkage chemistry can also determine the extent of π-electron delocalization across the framework. Fu *et al.* prepared the cyano-vinylene-linked **Bpy-sp** ^**2**^ **c-COF** through Knoevenagel condensation of 1,3,6,8-tetrakis(4-formylphenyl)pyrene (**TFPPy**) with 5,5′-bis(cyanomethyl)-2,2′-bipyridine. Relative to polarized imine linkages, the olefinic C=C connectivity establishes a more continuously conjugated 2D backbone and provides periodically arranged bipyridine sites for post-synthetic coordination (Figure 5c). The framework adopts an eclipsed AA-stacked structure according to Pawley refinement of the PXRD data, with one-dimensional (1D) diamond-shaped pores of ∼2.4 nm and a Brunauer–Emmett–Teller (BET) surface area of 432 m^2^·g^−1^. Treatment with [Re(CO)_5_Cl] generated **Re-Bpy-sp** ^**2**^ **c-COF** containing 18 wt% Re, corresponding to coordination of approximately half of the available bipyridine sites. FT-IR bands at 1900–2024 cm^−1^ were assigned to the retained [Re(CO)_3_Cl] unit, while XPS supported a Re–bipyridine coordination environment analogous to that of molecular [Re(bpy)(CO)_3_Cl]. Re incorporation extended the optical absorption edge from 589 to 694 nm and markedly altered the excited-state dynamics. Transient-absorption spectroscopy showed that the short-lived state of pristine **Bpy-sp** ^**2**^ **c-COF** (*t*_50_% ≈ 5 ps at 550 nm) was replaced by a substantially longer-lived charge-separated state in **Re-Bpy-sp** ^**2**^ **c-COF** (*t*_50_% ≈ 200 ps), accompanied by quenching of the delocalized 640-nm emissive state and formation of a long-lived non-emissive population. DFT calculations placed the HOMO predominantly on the pyrene donor and the LUMO on the Re–bipyridine acceptor, supporting photoinduced framework-to-catalyst e^−^ transfer. This mechanism differs from direct excitation of the triplet metal-to-ligand charge-transfer (^3^MLCT) state of homogeneous [Re(bpy)(CO)_3_Cl], illustrating how incorporation into an extended conjugated framework can alter the excitation and e^−^-delivery pathway of an otherwise molecularly related catalytic unit. Under visible-light irradiation (λ > 420 nm) with TEOA as the sacrificial donor, **Re-Bpy-sp** ^**2**^ **c-COF** produced CO at 1,040 μmol·g^−1^·h^−1^ with 81% selectivity, whereas the homogeneous Re complex deactivated within approximately 3 h; its activity also exceeded that of the compared Re-modified imine triazine COFs (∼750 μmol·g^−1^·h^−1^). Addition of [Ir(dF(CF_3_)ppy)_2_(dtbpy)]PF_6_ as an external photosensitizer increased the CO evolution rate to 1,400 μmol·g^−1^·h^−1^ with 86% selectivity. An amorphous analog exhibited a substantially lower turnover number (TON = 2.3 *vs*. 12.9 for the crystalline COFs), supporting an important role for structural order and accessible porosity in productive charge transfer and catalysis. In addition, photodeposition of Pt on **Re-Bpy-sp** ^**2**^ **c-COF** introduced a second catalytic function and enabled tunable syngas production, with the CO_2_:H_2_ ratio varying from approximately 4:1 to 1:10 as the Pt loading was changed. These results show that replacing polarized imine connectivity with an extended *sp* ^2^-carbon linkage can influence light absorption, charge-transfer lifetime and communication between the framework and Re catalytic center, while the modular pore architecture permits incorporation of additional catalytic sites for controlling product composition (Fu et al., 2020).

The same general principle has been extended to sacrificial-agent-free CO_2_ reduction coupled to H_2_O oxidation. Cheng *et al.* reported **viCOF-bpy-Re**, a chemically robust Re-containing COF constructed from 2,4,6-trimethyl-1,3,5-triazine and 5,5′-bis(formyl)-2,2′-bipyridine through fully conjugated vinylene (–C=C–) linkages (Figure 5d). Post-synthetic metalation produced a Re loading of 13.5 wt%, corresponding to approximately 40% of the bipyridine sites. The resulting material combines Re–bipyridine centers for CO_2_-to-CO reduction with triazine-containing framework domains that participate in the oxidative half-reaction. The low-polarity vinylene-linked conjugated backbone facilitates intraframework charge redistribution, and femtosecond transient-absorption measurements showed that Re coordination generated a charge-separated state with a lifetime of approximately 150 ps, consistent with transfer of photogenerated e^−^ toward Re-containing sites while oxidative equivalents remain associated with the triazine-rich framework. Under visible-light irradiation in H_2_O as the reaction medium and without sacrificial electron donors, **viCOF-bpy-Re** produced CO at 190.6 μmol g^−1^ h^−1^ with ∼100% selectivity and O_2_ at 90.2 μmol·g^−1^·h^−1^. Over 50 h of continuous irradiation, the cumulative yields reached 9,530 μmol·g^−1^ CO and 4,510 μmol·g^−1^ O_2_. The near 2:1 CO_2_ stoichiometry is consistent with coupling the two-electron CO_2_-to-CO reduction with the four-hole H_2_O-oxidation process. This system therefore demonstrates how hydrolytically robust, π-conjugated connectivity and spatially integrated reduction and oxidation sites can be combined within one crystalline framework to sustain overall photocatalytic CO_2_ conversion without a sacrificial donor (Cheng et al., 2023).

#### 3.2.2 Phenanthroline-based COF

Phenanthroline provides a more conformationally preorganized *N*,*N*-chelating environment than 2,2′-bipyridine because its two pyridyl units are locked within a rigid, fused aromatic scaffold, reducing rotational freedom while extending π conjugation.

Ai *et al.* exploited these features in **PAD-COF**, a 1D imine-linked framework synthesized from 4,4′-(1,10-phenanthroline-2,9-diyl)bis[benzaldehyde] (**PBA**) and 5′,5″-bis(4-aminophenyl)-[1,1′:3′,1″:3″,1‴-quaterphenyl]-4,4‴-diamine (**BAD**) (Figure 6). Unlike layered 2D COFs, where extensive interlayer π stacking can restrict access to buried coordination sites, **PAD-COF** forms an AA-stacked, ribbon-like architecture with accessible phenanthroline units along the framework. Pawley refinement gave an *R*_wp_ of 2.94%, with a calculated pore size of 16.36 Å and a BET surface area of 241.8 m^2^ g^−1^. Post-synthetic coordination of Co^2+^ to the phenanthroline sites generated a series of **Co**_**x**_ **SAS/PAD-COF** (*x* = 5, 10, 20, 40 and 80 mg CoCl_2_·6H_2_O), among which **Co**_**40**_ **SAS/PAD-COF**, containing 2.98 wt% Co, showed the highest photocatalytic activity. AC-HAADF-STEM, XANES and EXAFS supported atomically dispersed Co^2+^ species, with a dominant first-shell Co–N contribution at ∼1.7 Å, an average coordination number of ∼6.1 and no detectable Co–Co scattering. Co incorporation substantially modified both the electronic structure and interfacial kinetics of the framework: the optical band gap decreased from 2.80 to 2.72 eV, the conduction-band potential shifted to −1.90 V *vs*. SHE, and the average time-resolved photoluminescence lifetime increased from 1.55 to 1,028.14 ns Consistent with improved carrier transport, the interfacial charge-transfer resistance decreased from 126.25 to 9.01 kΩ, while CO_2_ uptake increased modestly after Co incorporation. Calculations further indicated that CO_2_ adsorbed at the Co site in a bent configuration with an O–C–O angle of 141.27°, consistent with substantial activation of the otherwise linear molecule. The reported energetics for formation of the *COOH intermediate changed from an unfavorable value of 1.04 eV on **PAD-COF** to a thermodynamically favorable value of −0.93 eV on **CoSAS/PAD-COF**, identifying stabilization of the early protonation intermediate as a major consequence of Co coordination. Under visible-light irradiation, **Co**_**40**_ **SAS/PAD-COF** produced CO at 3,091 μmol·g^−1^·h^−1^ with 93% selectivity, corresponding to a 43.7-fold increase over metal-free **PAD-COF**. The decrease in activity at higher Co loading further indicates that maximizing the number of metal sites is not sufficient; their dispersion and coupling to the photoactive framework must also be maintained. More broadly, this study shows that reducing framework dimensionality can offer an alternative way to control metal-site accessibility and charge transport, complementing the electronic and interlayer-engineering strategies developed for 2D COFs (Ai et al., 2024).

**FIGURE 6:**
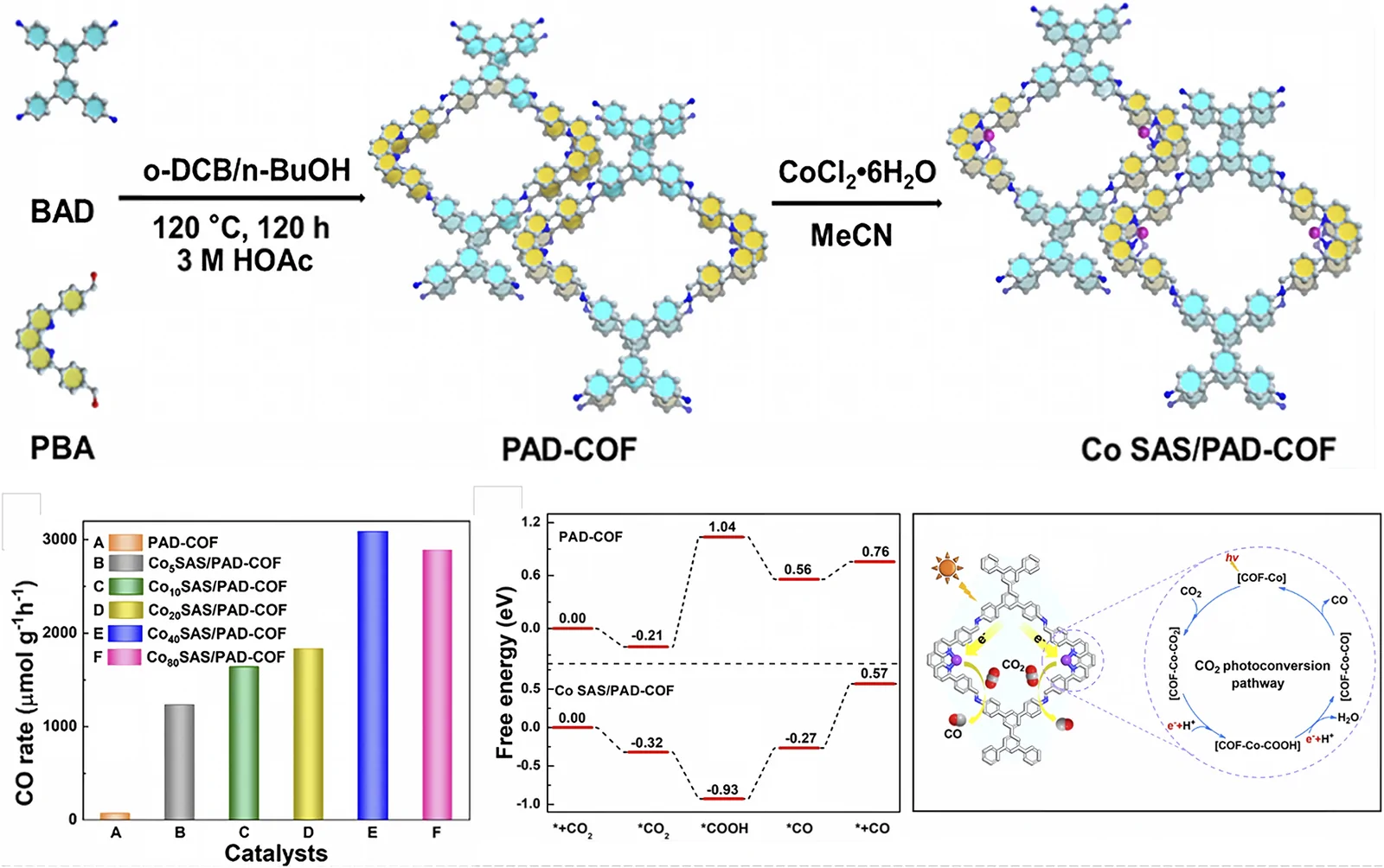
Synthetic scheme of **PAD-COF** and **CoSAS/PAD-COF**, average rate of CO generation over **PAD-COF** and **Co**_**x**_ **SAS/PADCOF**, calculated potential energy profile for photocatalytic CO_2_ to CO conversion on **CoSAS/PAD-COF** and **PAD-COF**, the mechanism for the photoconversion of CO_2_ into CO on **CoSAS/PAD-COF**. Reproduced with permission (Ai et al., 2024). Copyright 2024, Wiley-VCH GmbH.

#### 3.2.3 Catechol based COF

Zhang *et al.* further demonstrated that rational design of chelating motifs within COF precursors allows post-synthetic metalation to afford atomically dispersed Co–O_4_ sites, achieving coordination precision comparable to pre-metalated metallolinkers (Figure 7). Using 2,3-dihydroxyterephthalaldehyde (**2,3-DHTA**) as the aldehyde building block, they constructed a catechol-functionalized COF that, upon Co^II^ incorporation, yielded **Co-2,3-DHTA-COF** with a distinctive Co–O_4_ coordination geometry. For comparison, **Co-TP-COF** with a Co–O_3_N environment was also synthesized. EXAFS analysis unambiguously resolved the distinct local coordination environments around the Co centers in these two isostructural frameworks. Mechanistic studies revealed that these subtle variations in coordination microenvironments critically dictated reaction energetics: the rate-determining CO_2_ binding step at the Co–O_4_ site required significantly less energy (Δ*G* = 0.21 eV) than H_2_O displacement and CO_2_ adsorption at the Co–O_3_N site (Δ*G* = 0.50 eV). Consequently, **Co-2,3-DHTA-COF** delivered a remarkable CO production rate of 18,000 μmol·g^−1^·h^−1^, substantially outperforming its Co–O_3_N counterpart (11,600 μmol·g^−1^·h^−1^). This work elegantly illustrates how programmable modulation of metal coordination environments in post-synthetically metalated COFs enables precise tuning of structure–property relationships for high-efficiency CO_2_ photoreduction (Zhang Q. et al., 2023).

**FIGURE 7:**
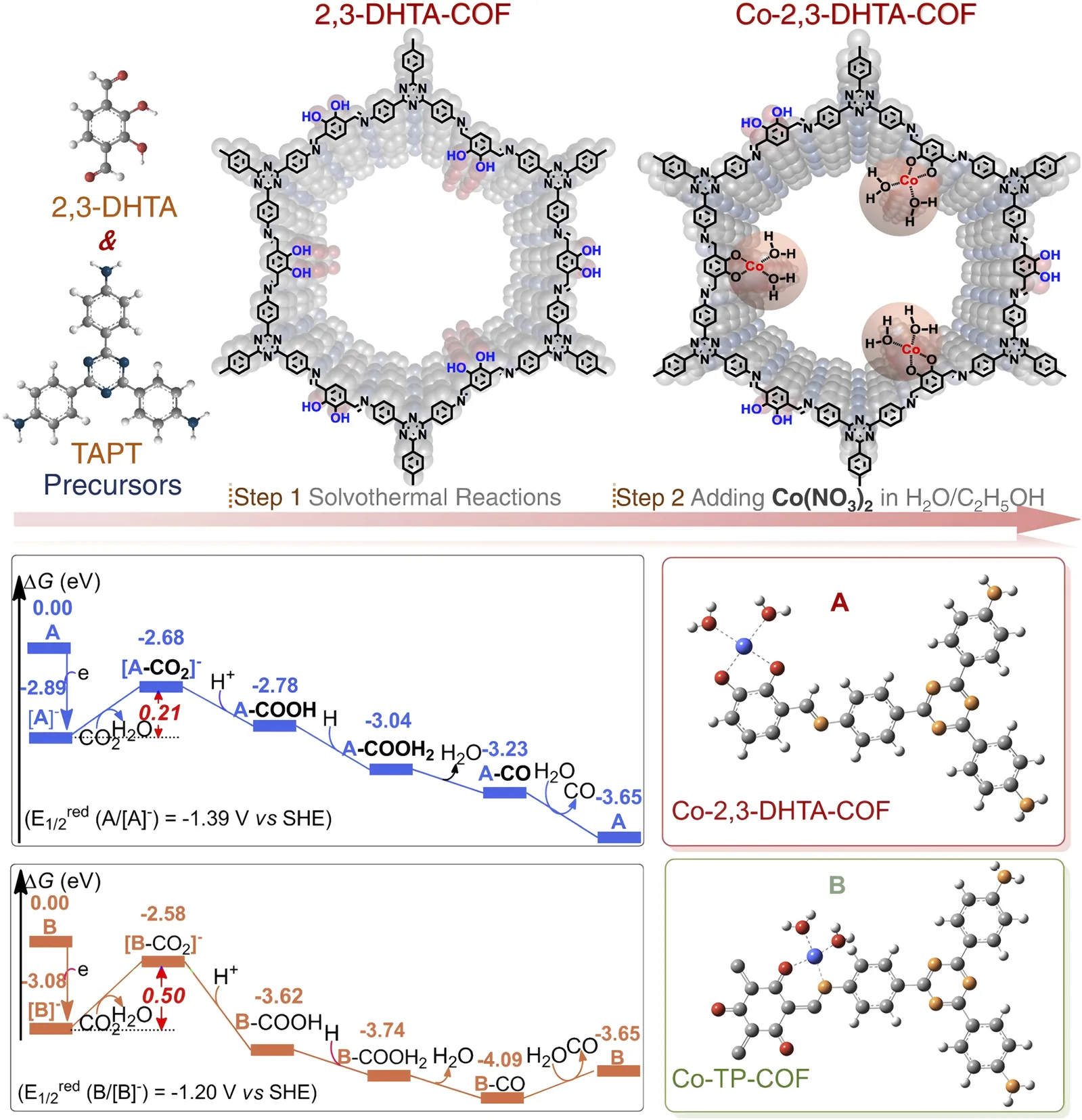
Schematic illustration of the synthesis of **2,3-DHTA-COF** and **Co-2,3-DHTA-COF** as typical examples, gibbs energy profiles of the CO_2_RR to CO over **Co-2,3-DHTA-COF** and **Co-TP-COF**. Reproduced with permission (Zhang Q. et al., 2023). Copyright 2023, springer nature.

## 4 Summary and outlook

Metallolinker-assembled COFs represent a significant conceptual advance in bridging molecular and heterogeneous catalysis for PCO_2_RR. By integrating well-defined coordination sites into crystalline porous scaffolds, this strategy offers a unique platform to decipher structure–activity relationships. However, the field is currently transitioning from proof-of-concept demonstrations to addressing fundamental limitations that hinder practical application.

### 4.1 Critical challenges

Despite notable progress, several intrinsic challenges specific to metallolinker-COF platforms remain unresolved:

*Fidelity of the Coordination Microenvironment:* A central premise of metallolinker COFs is the atomic precision of the catalytic site. However, maintaining the integrity of the pre-designed coordination geometry during framework polymerization or post-synthetic metalation is non-trivial. Framework constraints, residual solvents, or axial ligands can subtly alter the metal center’s electronic structure, creating a discrepancy between the intended molecular complex and the actual active site in the solid-state lattice.

*Electronic Coupling versus Site Isolation:* Covalently integrating metallolinkers into a conjugated framework creates strong electronic coupling between the metal site and the light-harvesting backbone. While this facilitates charge transfer, it complicates the deconvolution of photocatalytic performance: it is often difficult to distinguish whether activity enhancements stem from the intrinsic property of the metallolinker or from framework-mediated exciton dynamics. Excessive electronic delocalization may also dilute the unique molecular identity of the active site.

*Limited C*_*2+*_ *Product Selectivity:* Current metallolinker COFs predominantly yield C_1_ products (CO, HCOOH). The formation of C_2+_ hydrocarbons requires precise control over *CO intermediate adsorption and C–C coupling at adjacent metal centers. Designing asymmetric dual-metal sites with optimal charge polarization to stabilize dimeric intermediates remains a formidable synthetic and conceptual challenge that is rarely addressed in existing COF architectures.

*Long-Term Operational Stability:* The stability of metallolinker COFs under prolonged illumination and in aqueous or oxidative environments is a major concern. Issues include metal leaching from labile coordination sites, hydrolysis of dynamic covalent linkages (*e.g.*, imine bonds), and photocorrosion of the metal centers. The lack of standardized long-term stability tests under realistic conditions (*e.g.*, flue gas, natural sunlight) further obscures the practical viability of these materials.

*Reliance on Sacrificial Agents:* Most high-performance metallolinker COFs depend on sacrificial electron donors to suppress charge recombination. This dependence masks the intrinsic efficiency of the catalyst and precludes a true solar-to-fuel cycle. Designing systems where the metallolinker or framework functional groups can simultaneously drive both reduction and oxidation half-reactions without external sacrificial agents remains an elusive goal.

### 4.2 Future opportunities

Looking forward, several concrete directions can propel metallolinker COFs from promising prototypes to practically viable PCO_2_RR platforms:

*Rational Linkage Design*. Moving beyond traditional imine linkages toward more robust and electronically tunable connections (*e.g.*, olefin, *sp* ^2^C, dioxin) to enhance stability and modulate electronic communication with metal center. Linkage chemistry deserves particular attention because it simultaneously controls hydrolytic stability, interlayer electronic coupling and the polarity of the D–A pair, as demonstrated by the reversal of interlayer coupling in isomeric imine-linked COFs.

*Heterobimetallic Engineering and C*_*2+*_ *Formation*. Systematic exploration of asymmetric dual-metal sites to create tailored local charge distributions capable of stabilizing the dimeric intermediates required for C–C coupling. Because C_2+_ formation competes with CO desorption, it demands not only adjacent sites but also a site separation and an intermediate binding strength that are optimal for dimerization; single-site frameworks are inherently poorly suited to this requirement, and the design of crystalline frameworks with precisely positioned dual sites remains the least explored area of metallolinker chemistry. Greater attention should also be given to the possible involvement of framework-derived carbon in the measured C_2+_ yield, which must be excluded by ^13^CO_2_ labeling and carbon-balance analysis.

*Computational and Data-Driven Design*. First-principles screening of linker–metal combinations, combined with machine-learning prediction of adsorption energies and barrier heights, can narrow the enormous design space before synthesis. Full free-energy landscape calculations for competing pathways (the *OCHO *versus* *COOH branch, and *CO hydrogenation *versus* desorption) should become a standard complement to experimental selectivity data, and descriptor-based correlations between coordination geometry and product selectivity are needed to move beyond case-by-case optimization.

*Operando and Time-Resolved Mechanistic Studies*. Advanced *in situ*/operando spectroscopy should be used to monitor the dynamic evolution of metal oxidation states, coordination environments and intermediate coverages under working conditions, providing direct feedback for rational design. Time-resolved optical and X-ray methods are particularly important for establishing whether activity originates from the metallolinker itself or from framework-mediated exciton and charge-transfer dynamics, a distinction that steady-state measurements cannot resolve.

*Device Integration and Photon Utilization*. Translating powder photocatalysis into practical systems will require reactor and device engineering rather than catalyst optimization alone: immobilization of COF photocatalysts on monolithic supports or membranes to avoid light scattering and to simplify catalyst recovery, gas-phase or three-phase (gas–liquid–solid) reactors for improved CO_2_ mass transport, and integrated photoelectrode configurations that separate the reduction and oxidation half-reactions spatially. Because most reported quantum yields are measured at a single wavelength and low photon flux, apparent quantum yields should be reported as a function of wavelength, together with solar-to-fuel efficiency under simulated sunlight. Improving light utilization further requires suppressing reflection and scattering losses and extending absorption into the near-infrared region.

*Scalable Fabrication*. Most metallolinker COFs are currently obtained as microcrystalline powders on milligram scales in sealed vessels. Scaling-up will require reproducible protocols for linker synthesis and metalation, gram-to-kilogram scale solvothermal or mechanochemical routes that retain crystallinity, and continuous or flow-based manufacturing compatible with film and membrane formats. Cost and sustainability metrics, including the abundance and toxicity of the metal employed and the solvent burden of the synthesis, should be reported alongside photocatalytic performance.

*Performance under Realistic Feeds and Standardized Benchmarking*. Practical deployment requires evaluation under conditions that differ substantially from the idealized laboratory setup: dilute CO_2_ (5%–15%) in the presence of O_2_, SO_2_ and H_2_O vapor, ambient rather than saturated CO_2_ pressure, and continuous illumination over hundreds of hours. Long-term stability must be quantified with standard protocols that report the turnover number, the fraction of metal retained and the fate of the framework, rather than the crude “no obvious activity loss after four cycles” statement that is currently the norm. Establishing community-accepted benchmarking conditions would make the rapidly growing body of metallolinker-COF results genuinely comparable.

In conclusion, while metallolinker COFs have demonstrated the feasibility of merging molecular precision with heterogeneous robustness, overcoming the challenges of site fidelity, product selectivity and operational stability—supported by computational design, operando characterization and rational reactor engineering—will be crucial for their evolution from laboratory curiosities to practical solar fuel technologies.
